# Acylsulfonamide safety-catch linker: promise and limitations for solid–phase oligosaccharide synthesis

**DOI:** 10.3762/bjoc.8.232

**Published:** 2012-11-26

**Authors:** Jian Yin, Steffen Eller, Mayeul Collot, Peter H Seeberger

**Affiliations:** 1 Department of Biomolecular Systems, Max Planck Institute of Colloids and Interfaces, Am Mühlenberg 1, 14776 Potsdam, Germany; 2 Freie Universität Berlin, Institut für Chemie und Biochemie, Arnimallee 22, 14195 Berlin, Germany

**Keywords:** glycosaminoglycans (GAGs), glycosylation, resins, safety-catch linker, solid-phase synthesis

## Abstract

Safety-catch linkers are useful for solid-phase oligosaccharide synthesis as they are orthogonal to many common protective groups. A new acylsulfonamide safety-catch linker was designed, synthesized and employed during glycosylations using an automated carbohydrate synthesizer. The analysis of the cleavage products revealed shortcomings for oligosaccharide synthesis.

## Findings

**Figure 1:**
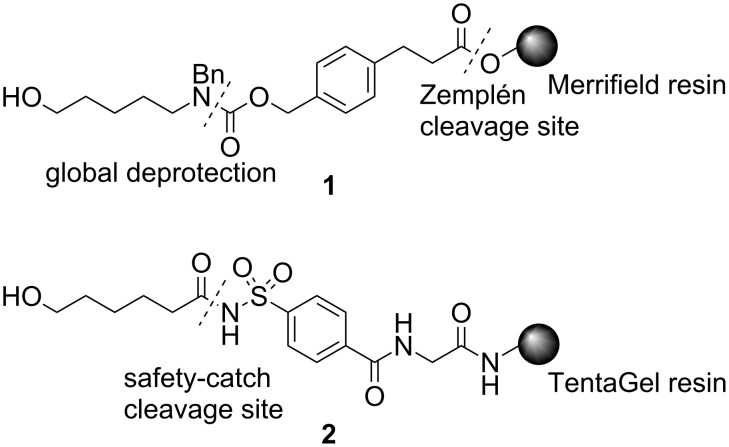
Structures of two novel linkers on different resins.

Solid-phase oligosaccharide synthesis [1–2] has been automated [3–5] to rapidly assemble complex oligosaccharides. Key to the success of solid-phase syntheses is the linker that connects the first carbohydrate building block to the solid support [6]. This linker has to remain stable throughout oligosaccharide synthesis but must be cleaved at the end of the reaction sequence to release the oligosaccharide and reveal a functional group for ready conjugation to array surfaces and carrier proteins. Since the first successful automated system for solid-phase oligosaccharide synthesis was introduced [7], alternative linker strategies have been explored [8–12]. Recently, a bifunctional amino–ester linker **1** [13–14] (Figure 1) has been developed, which can be readily cleaved from the resin by basic methanolysis. The released chromophore-containing part, such as aromatic benzyl ether protecting groups, facilitates the purification of the synthetic oligosaccharides by HPLC. Final deprotection of the product provides a terminal amine at the reducing end of the oligosaccharide for the fabrication of carbohydrate microarrays.

An acylsulfonamide safety-catch linker **2** (Figure 1), was developed in combination with TentaGel resin to provide orthogonality to temporary ester protecting groups. This linker was employed in the successful synthesis of a sialyl LewisX tetrasaccharide [15] and a sialyl Tn antigen [16]. In the search for a linker suitable for the solid-phase synthesis of complex glycosaminoglycans (GAGs) [17–18], we designed a new acylsulfonamide safety-catch linker that combined the advantageous features of linker **1**, the amino–ester bifunctional linker, and linker **2**, the safety-catch linker, to create a connection to the solid support that remains stable under conditions for cleaving temporary ester protective groups. Furthermore, the safety-catch linker should enable a variety of different reaction conditions on solid support since cleavage only occurs following preactivation. Thus, different modification reactions, such as Staudinger reduction, ester saponification or sulfation, necessary for the synthesis of GAGs, can be performed on solid support in an automated carbohydrate synthesizer. In the process of evaluating the performance of this linker in solid-phase glycosylation reactions, the potential as well as some severe limitations became apparent.

**Scheme 1:**
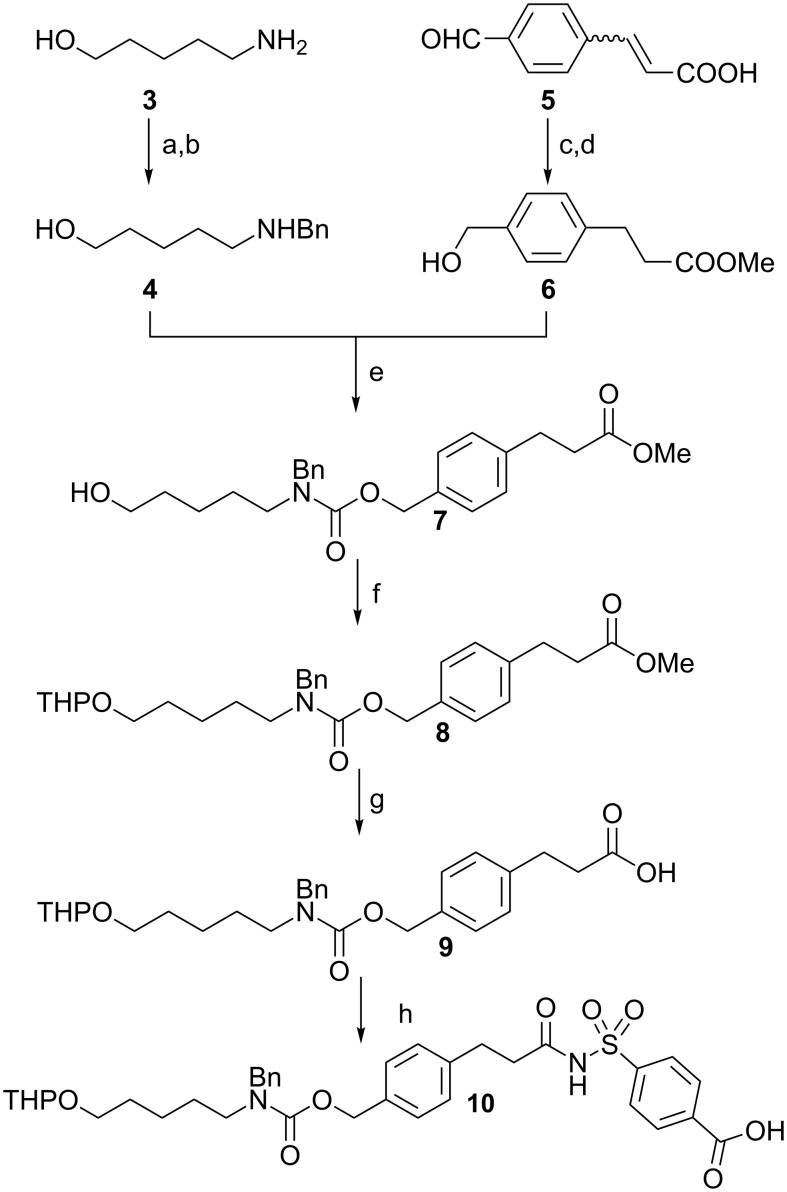
Synthesis of a new acylsulfonamide safety-catch linker. Reagents and conditions: (a) benzaldehyde, Na_2_SO_4_, DCM; (b) NaBH_4_, EtOH; 82% over two steps; (c) H_2_, 10% Pd/C, EtOH, DIPEA; (d) cat. H_2_SO_4_, MeOH, 88% over two steps; (e) DSC, Et_3_N, DCM/CH_3_CN, 85%; (f) DHP, *p*-TsOH·H_2_O, DCM, 86%; (g) LiOH·H_2_O, THF/H_2_O, reflux, 95%; (h) 4-sulfamoylbenzoic acid, DCC, DMAP, DCM/DMF, 60%.

Linker **10** is the newly designed acylsulfonamide safety-catch linker (Scheme 1). The safety-catch linkage to the resin permits methanolysis of temporary ester protecting groups. This linker can only be cleaved after activation with TMSCH_2_N_2_ or ICH_2_CN, afterwards, the subsequent aromatic protection of the masked amine enables UV detection during HPLC purification of the resultant oligosaccharide [13]. Finally, the terminal amine group revealed during final deprotection serves in the formation of glycoconjugates and glycan microarrays [19].

Synthesis of **10** relied on key intermediates **4** and **6** (Scheme 1). Monobenzylated amine **4** was prepared by reductive amination [20]. An established three-step synthesis starting with hydrocinnamic acid generated ester **6** with an overall yield of only 28% [21–22]. In contrast, when commercially available 4-formylcinnamic acid (**5**) served as starting material, ester **6** was prepared in just two steps and with an increased overall yield of 88% [23]. Reaction of alcohol **6** and disuccinimidyl carbonate (DSC) in the presence of NEt_3_ afforded a crude carbonate, which was smoothly reacted with amine **4** to provide carbamate **7** with 85% yield. Carbamate **7** was converted to the free acid **9** by simple protection to form THP ether **8** followed by saponification. Finally, coupling acid **9** with 4-sulfamoylbenzoic acid [15] afforded linker **10**.

**Scheme 2:**
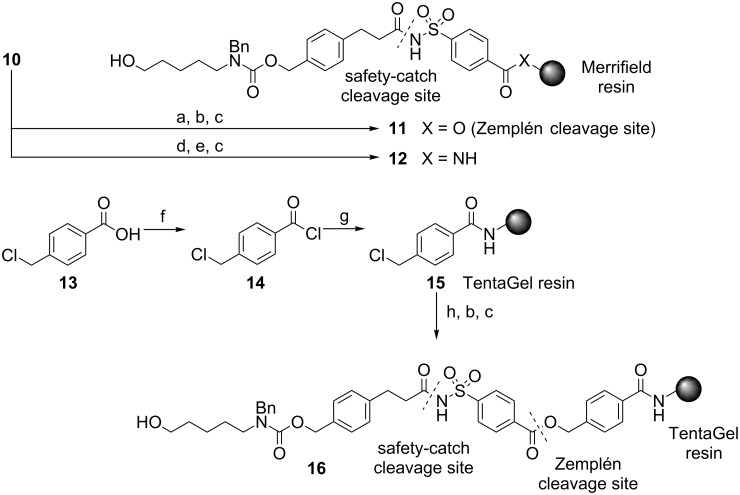
Functionalization of different resins. Reagents and conditions: (a) Cs_2_CO_3_, DMF, TBAI, Merrifield chloride resin; (b) CsOAc, DMF, TBAI; (c) *p*-TsOH·H_2_O, MeOH; (d) DIC, HOBt, DCM, Merrifield amine resin; (e) Ac_2_O, pyridine; (f) (COCl)_2_, DMF (cat), 99%; (g) TentaGel amine resin, DCM, pyridine, then Ac_2_O; (h) **10**, Cs_2_CO_3_, DMF, TBAI.

To support oligosaccharide synthesis, the safety-catch linker was first coupled to different resins (Scheme 2). In addition, since the activation and cleavage of safety-catch linkers is typically quite slow, a second, base-labile (Zemplén [24]) cleavage site was integrated to facilitate the fast release and analysis of glycosides. Linker-functionalized resin **11** contains both cleavage sites, and was accessed by coupling the cesium salt of linker **10** with Merrifield chloride resin prior to capping and deprotection (Scheme 2). A second resin **12**, containing only the safety-catch cleavage site, was created by installing linker **10** on Merrifield amine resin by treatment with DIC and HOBt (Scheme 2) [15]. After capping with acetic anhydride and removing the THP protecting group with *p*-TsOH·H_2_O, the functionalized resin **12** was ready for use. Finally, a third linker-functionalized resin **16** was created with TentaGel resin [15–16], which swells in water, because encouraging results have been achieved previously with this resin. 4-(Chloromethyl)benzoic acid (**13**) served as an additional spacer [25] between **10** and TentaGel amino resin to incorporate the Zemplén cleavage site. Treatment of acid **13** with oxalyl chloride yielded acyl chloride **14**, which, in turn, was coupled to linker **10** under the conditions established for the construction of resins **11** and **12**, and afforded modified TentaGel resin **16** with both cleavage sites (Scheme 2).

**Scheme 3:**
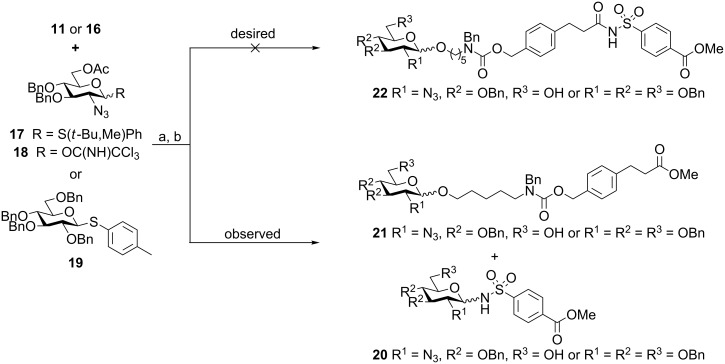
Glycosylation and cleavage reactions for analysis. Reagents and conditions: (a) automated glycosylations using **17**, **18** or **19**; (b) NaOMe, MeOH.

Functionalized resins **11** and **16**, both containing acylsulfonamide safety-catch linker **10** plus the Zemplén cleavage site, were employed in glycosylation reactions on a solid phase using an automated synthesizer [13], subsequently cleaved with NaOMe, and the products were analyzed by HPLC (Scheme 3, see Supporting Information File 1). Glycosylations were performed with either glucosamine thioglycoside **17** and glucosamine trichloroacetimidate **18** or perbenzylated thioglycoside **19**, which are both important building blocks for the synthesis of heparin and heparan sulfate. Three repetitions of a glycosylation using each three equivalents trichloroacetimidate **18** activated by TMSOTf were conducted, and followed by Zemplén cleavage. Similarly, three equivalents of thioglycosides **17** and **19** were added three times (triple coupling) for each glycosylation employing DMTST or NIS/TfOH as an activator. In both instances, surprisingly, only *N*-glycoside **20** (minor product) and glycosylated linker **21** (major product) rather than the desired product **22** were found (Scheme 3). *N*-Glycosidic sulfonamides were previously used during the synthesis of inhibitors of hepatocellular carcinoma cells [26]. This observation illustrates a limitation of the linker system since these undesired reactions result in the preactivation of the safety-catch linker, which can lead to cleavage in presence of nucleophiles. Similar results were observed when the experiments were repeated. The desired product **22** was detected in trace amounts only in the case of the coupling of thioglycoside **17** activated by NIS/TfOH to Merrifield resin **11**, as determined by HPLC (see Supporting Information File 1).

Based on these unexpected findings, we hypothesized that excess glycosylating agent may alkylate the sulfamyl group during glycosylation, such that subsequent cleavage provides products **20** and **21**. To test this, the amount of glycosylating agent was decreased during a reaction, which resulted in the production of glycosylated linkers **21** and **22**. It was evident that two unexpected reactions had occurred. First, the use of excess glycosylating agent, common practice for solid-phase-synthesis protocols, leads to activation of the sulfamyl group of the acylsulfonamide safety-catch linker, permitting cleavage at this location. This reaction had not been reported previously [15–16], because the architecture of this safety-catch linker means that the modified sulfamyl group remains on the resin after cleavage and release of the product, and this was not examined. As the new bifunctional resins **11** and **16** contain the additional Zemplén cleavage site, the product of this dominant but undesired reaction was evident. Second, the observation that reducing the concentration of the glycosylation agent caused an increase in the production of glycosylated linker **21**, implied that sodium methoxide may directly cleave safety-catch linkers without prior activation.

**Scheme 4:**
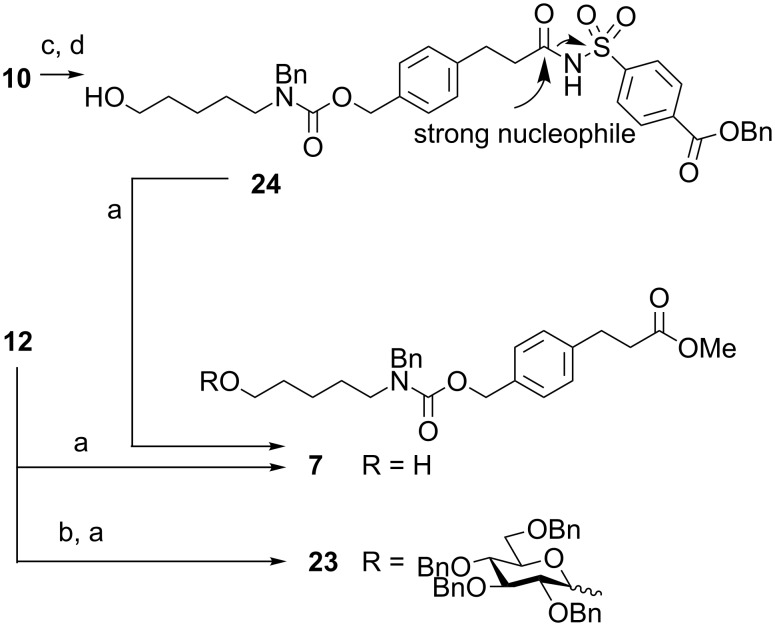
Further investigations of safety-catch linker. Reagents and conditions: (a) NaOMe, MeOH; (b) **19**, NIS, TfOH, DCM; (c) DCC, BnOH, DCM; (d) *p*-TsOH·H_2_O, MeOH; 55% over two steps.

To examine linker reactivity in more detail, additional automated glycosylations were performed by using thioglycoside **19** activated with NIS/TfOH, on Merrifield resin **12** containing only the safety-catch cleavage site (Scheme 4). The presence of glycosylated linker **23** as the main product confirmed that premature linker cleavage had occurred. Even nonglycosylated resin **12** was cleaved by treatment with sodium methoxide to afford linker **7** (Scheme 4). A possible explanation is provided by Unverzagt and co-workers [27], who reported, when using another acylsulfonamide safety-catch linker on a solid support, that capping the remaining amino group using acetic anhydride preactivates the sulfamyl group. To test this hypothesis, linker **10** was transformed by benzylation and deprotection to afford **24** prior to treatment with sodium methoxide (Scheme 4). Surprisingly, linker **7** was again isolated as the major product as determined by TLC and NMR, indicating that the nucleophilicity or basicity of NaOMe was responsible for cleavage. Other reagents, such as aqueous NaOH, hydrazine acetate, pyridine and benzylamine failed to cleave the linker. Even the strongly basic and weakly nucleophilic *t*-BuOK was not sufficient to induce premature cleavage. Thus, it is the nucleophilic action of sodium methoxide that is strong enough to induce cleavage of the safety-catch linker (Scheme 4).

In conclusion, we described a new acylsulfonamide safety-catch linker with an additional Zemplén cleavage site, designed for automated solid-phase synthesis of glycosaminoglycans. With this novel linker, inherent but previously unknown limitations of the safety-catch concept for solid-phase oligosaccharide synthesis were discovered. The sulfamyl group can be attacked by excess glycosylating agent to give the unexpected resin-bound *N*-glycoside, which may block additional reaction sequences and extension of the oligosaccharide [28]. In addition, sodium methoxide can directly cleave the sulfamyl group, prohibiting its use in conjunction with safety-catch linkers in general. While safety-catch linkers offer many attractive features for the solid-phase synthesis of complex molecules, particularly peptides, they should be used with great caution for oligosaccharide assembly.

## Supporting Information

File 1 Experimental section.

## References

[1] Seeberger P H, Haase W-C (2000). Chem Rev.

[2] Seeberger P H (2001). Solid Support Oligosaccharide Synthesis and Combinatorial Carbohydrate Libraries.

[3] Seeberger P H (2003). Chem Commun.

[4] Seeberger P H, Werz D B (2007). Nature.

[5] Seeberger P H (2008). Chem Soc Rev.

[6] Guillier F, Orain D, Bradley M (2000). Chem Rev.

[7] Plante O J, Palmacci E R, Seeberger P H (2001). Science.

[8] Love K R, Seeberger P H (2004). Angew Chem, Int Ed.

[9] Werz D B, Castagner B, Seeberger P H (2007). J Am Chem Soc.

[10] Codée J D C, Kröck L, Castagner B, Seeberger P H (2008). Chem–Eur J.

[11] Bindschädler P (2008). Different aspects of automeated heparin synthesis: de novo synthesis of building blocks, a new linker, and synthesis of heparin oligosaccharides.

[12] Czechura P, Guedes N, Kopitzki S, Vazquez N, Martin-Lomas M, Reichardt N-C (2011). Chem Commun.

[13] Kröck L, Esposito D, Castagner B, Wang C-C, Bindschädler P, Seeberger P H (2012). Chem Sci.

[14] Christ W, Kröck L, Plante O J, Castagner B, Seeberger P H (2010). Automated Oligosaccharide Synthesizer. WO Patent.

[15] Kanemitsu T, Wong C-H, Kanie O (2002). J Am Chem Soc.

[16] Kanemitsu T, Daikoku S, Kanie O (2006). J Carbohydr Chem.

[17] Yeung B K S, Chong P Y C, Petillo P A (2002). J Carbohydr Chem.

[18] Karst N A, Linhardt R J (2003). Curr Med Chem.

[19] Yin J, Seeberger P H (2010). Methods Enzymol.

[20] De Michelis C, Rocheblave L, Priem G, Chermann J C, Kraus J L (2000). Bioorg Med Chem.

[21] Arshady R, Atherton E, Clive D L J, Sheppard R C (1981). J Chem Soc, Perkin Trans 1.

[22] Párkányi C, Yeh Huang L S-C, Chu S G, Jeffries A T (1996). Collect Czech Chem Commun.

[23] Bolli M, Lehmann D, Mathys B, Mueller C, Nayler O, Velker J, Weller T (2006). Novel thiophene derivatives. PCT Int. Appl..

[24] Zemplén G, Kunz A (1923). Ber Dtsch Chem Ges.

[25] Wu X, Schmidt R R (2004). Eur J Org Chem.

[26] Colinas P A, Bravo R D (2005). Tetrahedron Lett.

[27] Mezzato S, Schaffrath M, Unverzagt C (2005). Angew Chem, Int Ed.

[28] de Paz J L, Kayser M M, Macchione G, Nieto P M (2010). Carbohydr Res.

