# Supplementary material for: Acylsulfonamide safety-catch linker: promise and limitations for solid–phase oligosaccharide synthesis
Source: Beilstein J Org Chem. 2012 Nov 26;8:2067–71. doi: 10.3762/bjoc.8.232 (PMC3511041; doi:10.3762/bjoc.8.232)
Supplement: File 1 — Experimental section. [file Beilstein_J_Org_Chem-08-2067-s001.pdf]

**Supporting Information**

**for**

# Acylsulfonamide safety-catch linker: promise and limitations for solid–phase oligosaccharide synthesis

Jian Yin[1,2,‡], Steffen Eller[1,2,‡], Mayeul Collot[1,2], and Peter H. Seeberger*[1,2]

Address: [1]Department of Biomolecular Systems, Max Planck Institute of Colloids and Interfaces, Am Mühlenberg 1, 14776 Potsdam, Germany and [2]Freie Universität Berlin Institut für Chemie und Biochemie, Arnimallee 22, 14195 Berlin, Germany

Email: Peter H. Seeberger - peter.seeberger@mpikg.mpg.de

[‡]Equally contributing authors

## Experimental section

**General information for chemical synthesis**: All chemicals used were reagent grade and were used as supplied, except where noted. All reactions were performed in oven-dried glassware under an inert atmosphere, unless noted otherwise. Reagent grade *N*,*N*-dimethylformamide (DMF) was dried over activated molecular sieves prior to use. Dichloromethane ($CH_2Cl_2$), toluene and tetrahydrofuran (THF) were purified by a Cycle-Tainer Solvent Delivery System, unless noted otherwise. Analytical thin-layer

chromatography (TLC) was performed on Merck silica gel 60 F254 plates (0.25 mm). Compounds were visualized by UV irradiation or by dipping the plate in a cerium sulfate ammonium molybdate (CAM) solution. Flash column chromatography was carried out by using forced flow of the indicated solvent on Fluka Kieselgel 60 (230–400 mesh). All automated glycosylations were performed on an automated oligosaccharide synthesizer prototype. LC–MS chromatograms were recorded on an Agilent 1100 Series spectrometer. Loading determination of functionalized resins was obtained by using a Shimadzu UV-MINI-1240 UV spectrometer. $^{1}$H, $^{13}$C NMR spectra were recorded on a Varian Mercury 400 (400 MHz) spectrometer in $CDCl_3$ with chemical shifts referenced to internal standards $CDCl_3$ (7.26 ppm, $^{1}$H; 77.0 ppm, $^{13}$C), unless stated otherwise. Splitting patterns are indicated as s, singlet; d, doublet; t, triplet; q, quartet; m, multiplet; brs, broad singlet for $^{1}$H NMR data. NMR chemical shifts (δ) are reported in ppm and coupling constants (*J*) are reported in Hz. High-resolution mass spectral (HRMS) analyses were performed by the MS-service in the Department of Organic Chemistry at the Free University Berlin. IR spectra were recorded on a Perkin-Elmer 1600 FTIR spectrometer.

**Synthesis of acylsulfonamide safety-catch linker**

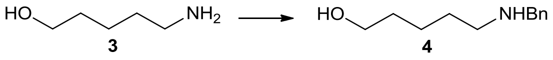


***N*-Benzyl-5-aminopentan-1-ol (4).** A solution of 5-aminopentanol **3** (18.8 g, 180 mmol) in $CH_2Cl_2$ (800 mL) was treated with PhCHO (20.4 mL, 200 mmol) and $Na_2SO_4$ (102.5 g, 1.8 mol) at room temperature, stirred for 18 h, filtered, and concentrated. The crude imine was solubilized in EtOH (110 mL) and treated with $NaBH_4$ (10.4 g, 273 mmol) at 0 °C. The reaction was stirred at 0 °C for 2 h, then at room temperature for 4 h, quenched with water (60 mL), and concentrated. The residue was solubilized in EtOAc (600 mL) and washed with 5% aqueous $NaHCO_3$ solution (2 × 250 mL). The aqueous layers were re-extracted once with EtOAc and the combined organic phases were dried over $MgSO_4$, filtered, and concentrated. After distillation of the impurity (BnOH) (140 °C, 1 mbar), *N*-benzyl-5-aminopentanol (**4**) [1] (28.9 g, 82%) was left as a colorless oil, which was applied directly to the next reaction.

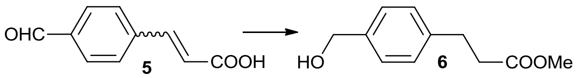


**Methyl 3-(4-(hydroxymethyl)phenyl)propanoate (6).** A mixture of 4-formylcinnamic acid (**5**) (4.00 g, 16.0 mmol) and Pd/C (500 mg, 10% Pd) in ethanol (170 mL) and DIPEA (5 mL) was stirred at room temperature for 6 h under a $H_2$ atmosphere. After filtration and evaporation, the crude product was dissolved in MeOH (160 mL), and 2 mL $H_2SO_4$ was added. The mixture was stirred overnight, followed by neutralization with 2 N NaOH solution and subsequent evaporation. The crude ester was purified by flash chromatography (*n*-hexane/ethyl acetate 3:1 → 2:1) to afford **6** [2] (3.8 g, 88%) as a white solid. $R_f$ 0.43 (cyclohexane/EtOAc 1:1). $^1$H NMR ($CDCl_3$, 400 MHz): δ 2.18 (s, 1H), 2.58 (t, *J* = 7.8 Hz, 2H), 2.91 (t, *J* = 7.8 Hz, 2H), 3.63 (s, 3H), 4.59 (s, 2H), 7.15 (d, *J* = 8.0 Hz, 2H), 7.24 (d, *J* = 8.0 Hz, 2H); $^{13}$C NMR ($CDCl_3$, 100 MHz): δ 30.5, 35.6, 51.6, 64.9, 127.2, 128.3, 138.9, 139.8, 173.3; IR ($CHCl_3$): 3368, 2924, 1738, 1438, 1366, 1198, 1015, 817 cm$^{-1}$; HRMS–ESI (*m*/*z*): [M + Na]$^+$ calcd for $C_{11}H_{14}O_3$, 217.0841; found, 217.0832.

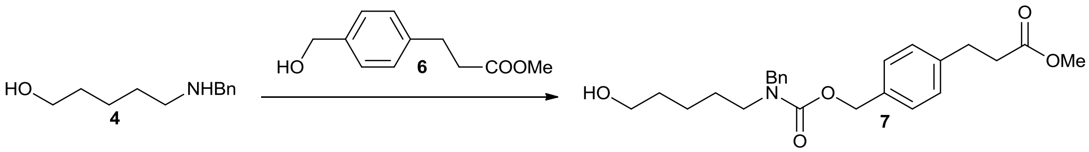


**Methyl 3-(4-((benzyl(5-hydroxypentyl)carbamoyloxy)methyl)phenyl)propanoate (7).** A solution of **6** (4.0 g, 20.5 mmol) in acetonitrile (110 mL) was treated with triethylamine (8.7 mL, 62 mmol) at room temperature then cooled to 0 °C. *N*,*N*'-Disuccinimidyl carbonate (DSC, 8.0 g, 31 mmol) was added. The solution was then warmed to 18 °C over 1 h, diluted with ethyl acetate (400 mL) and washed with saturated aqueous $NaHCO_3$ solution. The resultant aqueous layer was re-extracted once with ethyl acetate. The combined organic phases were dried over $MgSO_4$, filtered and concentrated in vacuo to afford the mixed carbonate, which was used without further purification. A solution of the crude carbonate in $CH_2Cl_2$ (45 mL) was added to a solution of **4** (8.0 g, 42 mmol) and triethylamine (7.3 mL, 52 mmol) in $CH_2Cl_2$ (110 mL) at 0 °C. The solution was stirred

for 50 min at room temperature, diluted with $CH_2Cl_2$ and washed with saturated aqueous $NaHCO_3$ solution. The aqueous layer was re-extracted twice with $CH_2Cl_2$. The combined organic phases were washed with brine, dried over $MgSO_4$, filtered and concentrated. Flash column chromatography on silica gel (cyclohexane/EtOAc 2:1 → 1:1) afforded **7** (7.4 g, 85%) as a colorless oil. $R_f$ 0.27 (cyclohexane/EtOAc 1:1); $^1$H NMR ($CDCl_3$, 400 MHz): δ 1.17–1.32 (m, 3H), 1.43–1.53 (m, 4H), 2.61 (br, 2H), 2.93 (br, 2H), 3.19–3.26 (m, 2H), 3.45–3.62 (m, 2H), 3.65 (s, 3H), 4.48 (d, $J$ = 6.8 Hz, 2H), 5.12 (d, $J$ = 11.2 Hz, 2H), 7.15–7.37 (m, 9H); $^{13}$C NMR ($CDCl_3$, 100 MHz): δ 22.8, 27.3, 27.8, 30.5, 32.2, 35.4, 46.0, 46.9, 50.0, 50.4, 51.5, 62.4, 66.9, 127.7, 128.0, 128.4, 128.6, 134.6, 134.7, 137.8, 140.2, 173.2; IR ($CHCl_3$): 3439, 3030, 2935, 2863, 2386, 1735, 1517, 1496, 1429, 1362, 1226, 1072 cm$^{-1}$; HRMS–ESI ($m/z$): $[M + Na]^+$ calcd for $C_{24}H_{31}NO_5Na$, 436.2100; found, 436.2092.

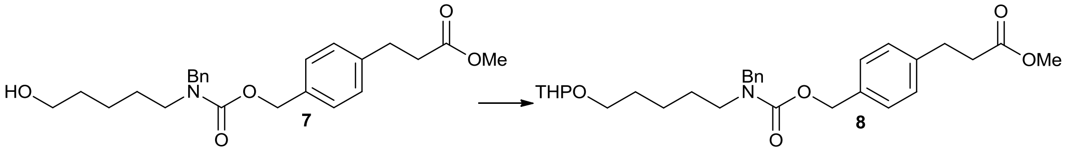


**Methyl 3-(4-((benzyl(5-*O*-tetrahydropyranyloxypentyl)carbamoyloxy)methyl)phenyl) propanoate (8).** To a mixture of **7** (6.8 g, 16.4 mmol) and 3,4-dihydro-2*H*-pyran (7.0 mL, 82.2 mmol, 5 equiv) in anhydrous $CH_2Cl_2$ (50 mL), pyridinium *p*-toluenesulfonate (2.1 g, 8.2 mmol, 0.5 equiv) was added and the solution was stirred overnight. Next, saturated $NaHCO_3$ solution was added and the resultant organic layer dried over $MgSO_4$, filtered and concentrated. Flash column chromatography on silica gel (cyclohexane/EtOAc 4:1 → 3:1) afforded **8** (7.1 g, 86%) as a colorless oil. $R_f$ 0.66 (cyclohexane/EtOAc 1:1); $^1$H NMR ($CDCl_3$, 400 MHz): δ 1.13–1.30 (m, 3H), 1.41–1.52 (m, 8H), 1.57–1.79 (m, 3H), 2.55 (br, 2H), 2.86 (br, 2H), 3.12–3.26 (m, 3H), 3.38–3.43 (m, 1H), 3.59 (s, 3H), 3.74–3.79 (m, 1H), 4.44 (t, $J$ = 12.4 Hz, 2H), 5.06 (d, $J$ = 10.6 Hz, 2H), 7.08–7.21 (m, 9H); $^{13}$C NMR ($CDCl_3$, 100 MHz): δ 19.7, 23.5, 25.5, 29.4, 30.6, 30.8, 35.6, 51.6, 62.4, 66.9, 67.0, 67.3, 77.2, 98.9, 127.2, 127.8, 128.1, 128.2, 128.4, 128.5, 137.9, 140.2, 173.2; IR ($CHCl_3$): 2939, 2865, 1737, 1723, 1517, 1496, 1454, 1227, 1034 cm$^{-1}$; HRMS–ESI ($m/z$): $[M + Na]^+$ calcd for $C_{29}H_{39}NO_6Na$, 520.2675; found, 520.2673.

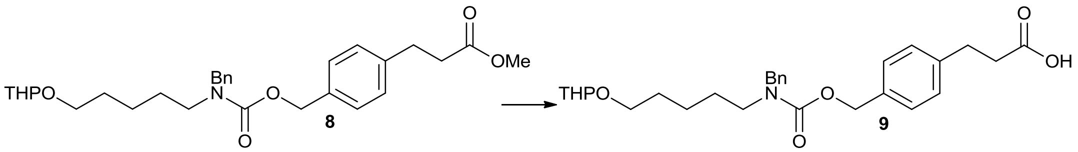


**3-(4-((Benzyl(5-*O*-tetrahydropyranyloxypentyl)carbamoyloxy)methyl)phenyl)propanoic acid (9).** To a solution of **8** (5.1 g, 10.2 mmol) in THF/water (60 mL, 4:1), LiOH·$H_2O$ (1.23 g, 30.7 mmol, 3 equiv) was added. The solution was stirred under reflux for 2 h and then cooled to room temperature. The solution was adjusted to pH 5–6 by using IR-120 ($H^+$). The mixture was then filtered and concentrated. Flash column chromatography on silica gel (DCM/MeOH 150:1 → 100:1) afforded **9** (4.8 g, 95%) as a light-yellow oil. $R_f$ 0.33 (DCM/MeOH 50:1); $^1$H NMR ($CDCl_3$, 400 MHz): δ 1.26–1.33 (m, 2H), 1.49–1.62 (m, 7H), 1.67–1.84 (m, 2H), 2.55 (br, 2H), 2.67 (br, 2H), 2.96 (d, *J* = 4.3 Hz, 2H), 3.20–3.40 (m, 3H), 3.48–3.53 (m, 1H), 3.64–3.68 (m, 1H), 3.82–3.87 (m, 1H), 4.50 (d, *J* = 8.8 Hz, 2H), 4.57 (t, *J* = 6.5 Hz, 1H), 5.14 (d, *J* = 10.0 Hz, 2H), 7.17–7.29 (m, 9H); $^{13}$C NMR ($CDCl_3$, 100 MHz): δ 19.5, 22.8, 25.4, 27.3, 27.8, 29.3, 30.3, 30.6, 35.4, 46.0, 47.0, 50.1, 50.4, 62.3, 67.0, 67.3, 98.7, 127.2, 127.7, 128.1, 128.4, 134.7, 137.8, 140.0, 156.2, 156.7, 177.3, 177.6; IR ($CHCl_3$): 3029, 2939, 2864, 1732, 1517, 1496, 1474, 1419, 1355, 1226, 1184, 1086, 1074, 1021, 972 $cm^{-1}$; HRMS–ESI (*m*/*z*): $[M + Na]^+$ calcd for $C_{28}H_{37}NO_6Na$, 506.2519; found, 506.2512.

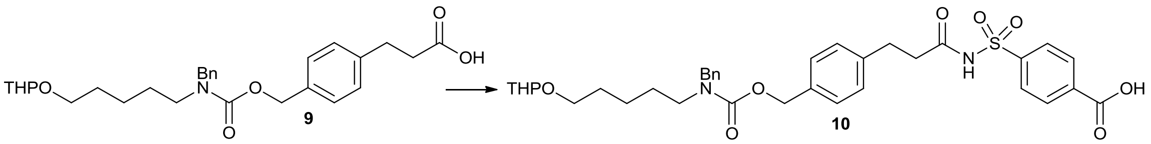


**4-(*N*-(3-(4-(((benzyl(5-((2*H*-tetrahydropyran-2-yl)oxy)pentyl)carbamoyl)oxy)methyl)phenyl)propionyl)sulfamoyl)benzoic acid (10).** To a solution of DCC (765 mg, 3.7 mmol) in anhydrous $CH_2Cl_2$ (10 mL), **9** (3.5 g, 7.2 mmol) was added at room temperature. The mixture was stirred for 3 h and then filtered to remove urea. The filtrate was added to a solution containing 4-sulfamoylbenzoic acid (804 mg, 4.0 mmol) in DMF (15 mL) at room temperature. The mixture was stirred overnight, then diluted with EtOAc, washed three times with brine, dried over $Na_2SO_4$, filtered, and concentrated. Flash column

chromatography on silica gel (DCM/MeOH/HOAc 150:1:0.05 → 100:1:0.05) afforded **10** (2.9 g, 60%) as a colorless yellow oil. $R_f$ 0.20 (DCM/MeOH 50:1); $^1$H NMR ($CDCl_3$, 400 MHz): δ 1.22–1.31 (m, 3H), 1.49–1.58 (m, 8H), 1.69–1.85 (m, 2H), 2.57 (br, 2H), 2.84 (d, *J* = 6.9 Hz, 2H), 3.20–3.35 (m, 3H), 3.52–3.69 (m, 3H), 3.84–3.92 (m, 1H), 4.47–4.58 (m, 3H), 5.07 (t, *J* = 16.9 Hz, 2H), 6.94–7.07 (m, 3H), 7.15–7.29 (m, 6H), 7.99–8.05 (m, 2H), 8.12–8.16 (m, 2H); $^{13}$C NMR ($CDCl_3$, 100 MHz): δ 19.4, 22.6, 23.2, 23.3, 25.3, 27.7, 29.2, 29.3, 29.8, 30.6, 31.7, 37.7, 46.2, 47.0, 50.1, 50.4, 62.3, 62.4, 62.5, 67.1, 67.2, 67.4, 98.9, 127.1, 127.3, 127.5, 128.0, 128.2, 128.5, 130.4, 134.3, 134.6, 137.4, 137.5, 139.5, 139.7, 134.2, 156.5, 157.0, 168.0, 170.7; IR ($CHCl_3$): 2939, 2865, 1672, 1454, 1354, 1172, 1021, 862 cm$^{-1}$; HRMS–ESI (*m*/*z*): $[M + Na]^+$ calcd for $C_{35}H_{42}N_2O_9SNa$, 689.2509; found, 689.2497.

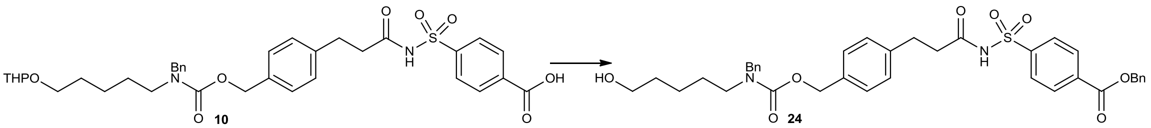


**Benzyl 4-(*N*-(3-(4-((benzyl(5-hydroxypentyl)carbamoyloxy)methyl)phenyl)propionyl)sulfamoyl)benzoate (24).** To a solution of DCC (46 mg, 0.22 mmol) and DMAP (6 mg, 0.05 mmol) in anhydrous $CH_2Cl_2$ (5 mL), **10** (286 mg, 0.43 mmol) was added with benzyl alcohol (0.18 mL, 1.72 mmol) at room temperature. The mixture was stirred for 8 h and filtered. The filtrate was evaporated to afford the crude product that was dissolved in MeOH (5 mL). *p*-TsOH·$H_2O$ (42 mg, 0.22 mmol) was added to the solution, which was then stirred for another 6 h, and concentrated. Flash column chromatography on silica gel (cyclohexane/EtOAc 2:1 → 1:1) afforded **24** (158 mg, 55%) as a colorless oil over two steps. $R_f$ 0.49 (cyclohexane/EtOAc 1:2); $^1$H NMR ($CDCl_3$, 400 MHz): δ 0.75–0.90 (m, 2H), 1.09–1.19 (m, 5H), 1.34–1.45 (m, 5H), 2.42 (t, *J* = 7.5 Hz, 2H), 2.73 (br, 1H), 3.10–3.19 (m, 2H), 3.35–3.52 (m, 2H), 4.40 (d, *J* = 12.6 Hz, 2H), 4.93–5.06 (m, 2H), 6.90–7.37 (m, 14H), 7.94 (dd, *J* = 1.8, 6.9 Hz, 2H), 8.08 (d, *J* = 8.4 Hz, 2H); $^{13}$C NMR ($CDCl_3$, 100 MHz): δ 14.1, 22.6, 27.2, 29.6, 32.1, 35.8, 37.9, 46.0, 46.7, 50.0, 50.4, 62.6, 67.1, 67.4, 127.1, 127.3, 127.6, 128.0, 128.2, 128.3, 128.5, 128.6, 130.3, 134.5, 135.3, 137.6, 142.7, 156.4, 156.8, 164.8, 170.4, 170.6; IR ($CHCl_3$): 3032, 2931, 2859,

1723, 1673, 1578, 1496, 1453, 1429, 1352, 1171, 1017, 958 cm$^{-1}$; HRMS–ESI (*m*/*z*): $[M + Na]^+$ calcd for $C_{37}H_{40}N_2O_8SNa$, 695.2403; found, 695.2408.

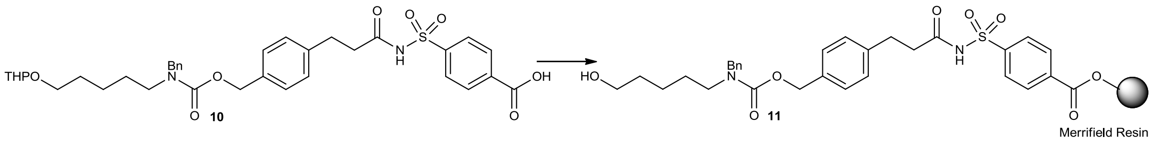


**Linker-functionalized Merrifield chlorine resin 11.** Merrifield chlorine resin (300 mg) was swollen by overnight incubation in $CH_2Cl_2$ with gentle rotation on a rotovap. The solvent was drained and a solution of **10** (1.5 equiv) in DMF (2 mL) was added to the resin followed by $Cs_2CO_3$ (2.0 equiv) and then TBAI (1.5 equiv). The solution was incubated at 60 °C overnight while rotating on the rotovap. The coupling reaction was repeated once. Subsequently, the resin was washed successively with DMF/Water (1:1), DMF, MeOH, $CH_2Cl_2$, MeOH, $CH_2Cl_2$ and then allowed to swell in $CH_2Cl_2$ for 1 h. The fully-swollen resin was placed in a flask with DMF (2 mL) and CsOAc (2.0 equiv) was added. The solution was allowed to rotate at 60 °C on the rotavap overnight. The capping reaction was repeated once. The capped, functionalized resin was washed successively with DMF/Water (1:1), DMF, MeOH, $CH_2Cl_2$, MeOH, $CH_2Cl_2$. Afterwards, the resin was incubated and shaken overnight in a 30 mM solution of *p*TsOH·$H_2O$ in MeOH. This THP removal reaction was repeated twice. The solution was then drained and the resin washed successively with $CH_2Cl_2$, MeOH and $CH_2Cl_2$ prior to drying in high vacuum.

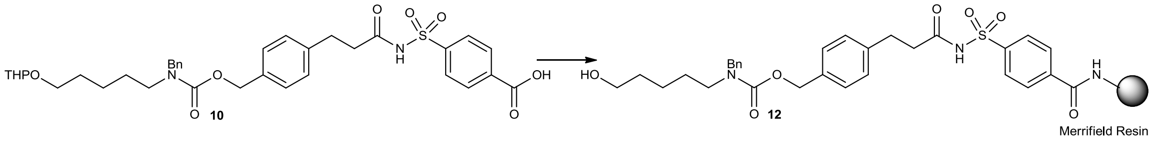


**Linker-functionalized Merrifield amino resin 12.** The Merrifield amino resin (300 mg) was swollen by overnight incubation in $CH_2Cl_2$. The solvent was drained and a solution of **10** (1.5 equiv) in DMF (2 mL) was added to the resin followed by addition of HOBt (6 equiv) and DIC (6 equiv). The resin solution was shaken overnight at room temperature. Subsequently, the coupling reaction was repeated once. The resin was then washed

successively with $CH_2Cl_2$, MeOH and $CH_2Cl_2$, and then capped with a solution of $CH_2Cl_2$/pyridine/$Ac_2O$ (2:1:1). The capping reaction was carried out one more time. The resin was then washed successively with $CH_2Cl_2$, MeOH, and $CH_2Cl_2$, and then with a 0.03 M solution of PTSA monohydrate in $CH_2Cl_2$ to remove pyridine. The linker-functionalized resin was then shaken overnight in a 30 mM solution of *p*TsOH·$H_2O$ in $CH_2Cl_2$. This THP removal reaction was repeated twice. The solution was drained and the resin was washed successively with $CH_2Cl_2$, MeOH and $CH_2Cl_2$ prior to drying under a vacuum.

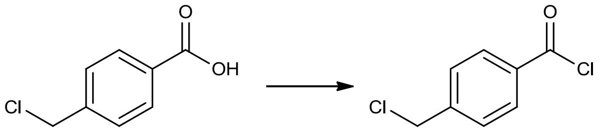


13 14

**(Chloromethyl)benzoyl chloride (14).** To a flask containing (chloromethyl)benzoic acid **13** (2.00 g, 11.76 mmol), oxalyl chloride (5 mL) was added under the control of a bubbler. Next, a drop of DMF was carefully added and the reaction was stirred at room temperature overnight. The solvents were evaporated to give 2.20 g (99%) of pure **14** [3] as a syrup. $^1$H NMR (400 MHz, $CDCl_3$): δ 8.12 (d, *J* = 8.4 Hz, 2H), 7.54 (d, *J* = 8.5 Hz, 2H), 4.63 (s, 2H).

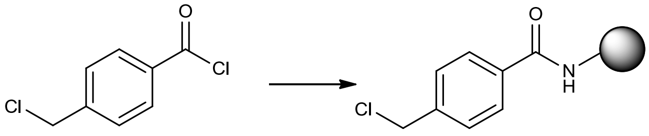


14 15

**Spacer-functionalized Merrifield amino resin 15.** The TentaGel amino resin (300 mg) was swollen by incubating in $CH_2Cl_2$ overnight. The solvent was drained and a solution of **14** (2.0 equiv) in $CH_2Cl_2$/pyridine (2 mL, 3:2) was added. The resin was shaken overnight at room temperature, and then the coupling reaction was carried out one more time. The spacer-functionalized resin was washed with $CH_2Cl_2$, MeOH and $CH_2Cl_2$, and then was capped with $CH_2Cl_2$/pyridine/$Ac_2O$ (2:1:1). The capping reaction was repeated once. The functionalized resin was then shaken overnight. Next a series of washes was

performed with $CH_2Cl_2$, MeOH, $CH_2Cl_2$, and then a 30 mM solution of *p*-TsOH·$H_2O$ in $CH_2Cl_2$ to remove pyridine, prior to drying in high vacuum

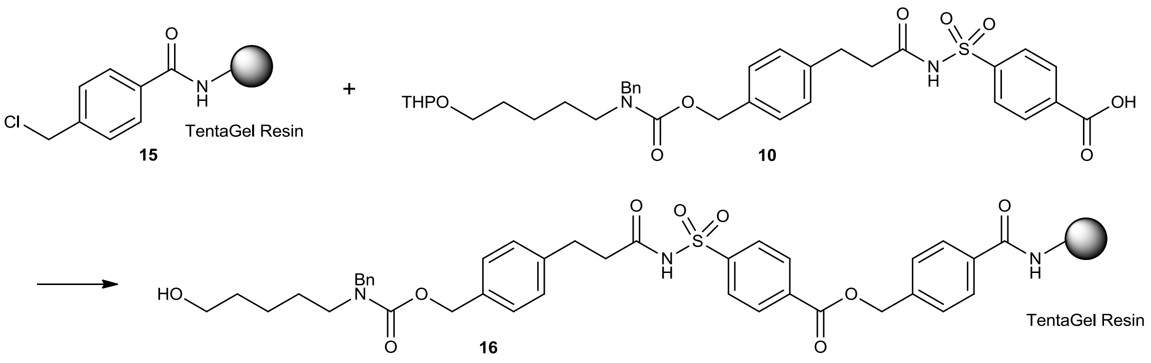


**Linker-functionalized Merrifield amino resin 16.** Functionalized TentaGel resin **15** (300 mg) was swollen by overnight incubation in $CH_2Cl_2$ with gentle rotation on a rotovap. The solvent was drained and a solution of **10** (1.5 equiv) in DMF (2 mL) was added to the resin followed by $Cs_2CO_3$ (2.0 equiv) and TBAI (1.5 equiv). The solution was incubated at 60 °C overnight in the rotovap, after which the coupling reaction was repeated once. Next, the resin was washed successively with DMF/water (1:1), DMF, MeOH, $CH_2Cl_2$, MeOH, $CH_2Cl_2$, and then allowed to swell in $CH_2Cl_2$ for 1 h. The fully-swollen resin was placed in a flask with DMF, and CsOAc (2.0 equiv) was added. The solution was rotated at 60 °C in the rotavap overnight. The capping reaction was repeated once. The functionalized resin was then washed successively with DMF/water (1:1), DMF, MeOH, $CH_2Cl_2$, MeOH, and $CH_2Cl_2$. The resin was then incubated overnight with shaking in a 30 mM solution of *p*-TsOH·$H_2O$ in $CH_2Cl_2$. After this, the THP removal reaction was repeated twice. The solution was drained and the resin was washed successively with $CH_2Cl_2$, MeOH and $CH_2Cl_2$ prior to drying in high vacuum.

**General method to determine the loading of functionalized resins 11, 12 and 16.**

By using a method adapted from the work of Gude and co-workers [4], dried functionalized resin (20–30 mg) was placed in a syringe equipped with a frit (5 mL syringe). $CH_2Cl_2$ (2–3 mL) was added to the syringe for 1 h to swell the resin, and then

drained. Next, 1 mL of $CH_2Cl_2$, 0.1 mL of pyridine, 100 mg of FmocCl, was added and the syringe was shaken for at least 6 h. The solution was drained and the resin was washed with $CH_2Cl_2$ (three times), MeOH (three times), then alternating washes of $CH_2Cl_2$ and MeOH, each three times. The resin was again swollen in $CH_2Cl_2$ and drained. A 2% (v/v) DBU in DMF solution (2 mL) was added to the syringe, which was shaken for at least 1 h. Finally, the solution was drained from the resin into a vial and retained.

For each solution obtained from **11**, **12** and **16**, 0.160 mL was added to a 10 mL graduated flask and diluted to 10 mL with acetonitrile. To calculate the loading of each resin, UV absorption at 304 nm was obtained, by using 2% (v/v) DBU in DMF solution (0.160 mL) diluted to 10 mL with acetonitrile as a blank control. The loading was calculated according to the following formula:

Loading [mmol/g]= A304 × 16.4/g resin

In this way at 304 nm loading was determined to be:
Functionalized resin **11** = 0.566 mmol/g of resin
Functionalized resin **12** = 0.249 mmol/g of resin
Functionalized resin **16** = 0.433 mmol/g of resin

**General procedures for automated syntheses**

Thioglycoside glycosylation - activation with NIS/triflic acid:

The chosen functionalized resin was loaded into the reaction vessel of the synthesizer. The temperature of the reaction vessel was adjusted to 25 °C and the resin was treated with successive washes of DMF, THF, 0.2 M acetic acid in $CH_2Cl_2$ and $CH_2Cl_2$ (six times, each wash with 2 mL for 25 s). For glycosylation, a solution of thioglycoside building blocks (**17** or **19**; 3 equiv or 1.1 equiv in 1 mL $CH_2Cl_2$) was delivered to the reaction vessel followed by the addition of 1 mL of activation solution (170 mM NIS and 17 mM triflic acid in dioxane/$CH_2Cl_2$ 1:1). After 15 min, the glycosylation solution was drained and the resin was washed once with 2 mL dioxane, followed by washes with 0.2 M acetic

acid in $CH_2Cl_2$ and $CH_2Cl_2$ successively (six times, each wash with 2 mL for 25 s). This glycosylation reaction was repeated twice.

Thioglycoside glycosylation - activation with DMTST:

The chosen functionalized resin was loaded into the reaction vessel of the synthesizer. The temperature of the reaction vessel was adjusted to 25 °C and the resin was washed with DMF, THF, 10% acetone in $CH_2Cl_2$ and $CH_2Cl_2$ successively (six times, each wash with 2 mL for 25 s). For glycosylation reactions the temperature was adjusted to −15 °C and a solution of thioglycoside building blocks (**17** or **19**; 3 equiv in 1 mL $CH_2Cl_2$) was delivered to the reaction vessel. The reaction was initiated by the addition of 1 mL of 181 mM DMTST solution (181 mM $Me_2S_2$ and 181 mM MeOTf in $CH_2Cl_2$). After 3 h the glycosylation solution was drained and the resin was washed with $CH_2Cl_2$ (six times, each wash with 2 mL for 25 s). This glycosylation reaction was repeated twice.

Trichloroacetimidate glycosylation - activation with TMSOTf:

The chosen functionalized resin was loaded into the reaction vessel of the synthesizer. The temperature of the reaction vessel was adjusted to 25 °C and the resin was washed with DMF, THF, 10% acetone in $CH_2Cl_2$ and $CH_2Cl_2$ successively (six times each with 2 mL for 25 s). The resin was washed with 2 mL of 17 mM triflic acid in $CH_2Cl_2$, and $CH_2Cl_2$ one time each, and the temperature was adjusted to −15 °C. For glycosylation, a solution of glycosyl trichloroacetimidate building block **18** (3 equiv in 0.75 mL $CH_2Cl_2$) was delivered to the reaction vessel followed by the addition of 0.25 mL of 50 mM TMSOTf in $CH_2Cl_2$. After 30 min the glycosylation solution was drained and the resin was washed with $CH_2Cl_2$ (six times with 2 mL for 25 s). The acidic prewash with triflic acid and the glycosylation reaction was repeated twice.

**Example of an experimental procedure**

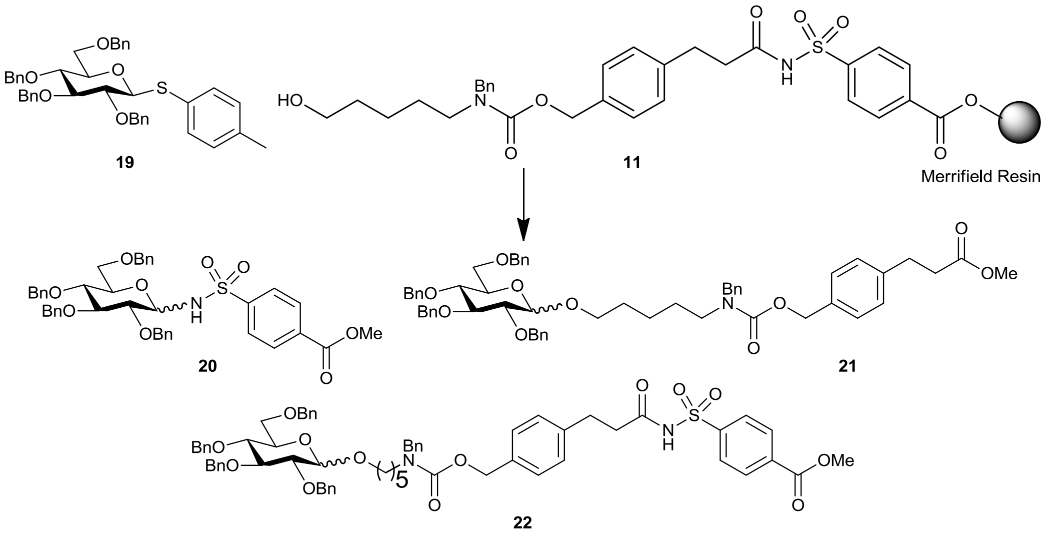


Automated glycosylation to safety-catch linker:

Functionalized resin **11** (50 mg, loading: 0.566 mmol/g, 28.3 μmol) was loaded into the reaction vessel of the synthesizer. The temperature of the reaction vessel was adjusted to 25 °C and the resin was treated with successive washes of DMF, THF, 0.2 M acetic acid in $CH_2Cl_2$ and $CH_2Cl_2$ (six times, each wash with 2 mL for 25 s) and one time with activation solution (170 mM NIS and 17 mM triflic acid in dioxane/$CH_2Cl_2$ 1:1; 2 mL for 25 s) and $CH_2Cl_2$ (2 mL for 25 s). For glycosylation, a solution of building block **19** (55 mg, 3 equiv, 84.9 μmol) was delivered to the reaction vessel followed by the addition of 1 mL of activation solution (170 mM NIS and 17 mM triflic acid in dioxane/$CH_2Cl_2$ 1:1). After 15 min, the glycosylation solution was drained and the resin was washed once with 2 mL dioxane, followed by washes $CH_2Cl_2$ (six times, each wash with 2 mL for 25 s) and one time with activation solution (170 mM NIS and 17 mM triflic acid in dioxane/$CH_2Cl_2$ 1:1; 2 mL for 25 s) and $CH_2Cl_2$ (2 mL for 25 s). This glycosylation reaction was repeated twice and the resin was finally washed with $CH_2Cl_2$ (six times with 2 mL for 25 s).

For the Zemplén cleavage the resin was removed from the reaction vessel of the synthesizer and was put into a syringe equipped with a filter frit. The solid support was swollen in $CH_2Cl_2$ and a NaOMe solution (0.25 M in MeOH, 0.5 mL) was added. The reaction was shaken at room temperature. After 10 h, the solution was collected and the resin was washed with $CH_2Cl_2$ and $CH_2Cl_2$/MeOH 1:1 (six times, each wash with 2 mL).

The solutions were combined and neutralized with IR-120 ($H^+$) and the solvents were removed in vacuo.

RPHPLC–MS analysis:

The crude product mixture was analyzed by RPHPLC–MS (column: Nucleosil C4 (120 mm × 5 mm); detection: UV detection at 209, 230 and 254 nm; eluents: water (+ 0.1% formic acid) and acetonitrile (+ 0.1% formic acid); gradient: isocratic at 10% for 5 min → 10% → 80% acetonitile in 30 min → 95% acetonitrile in 10 min; for chromatogram and mass spectra, please see below).

| compound | retention time [min] | *m*/*z* calcd for | obsd |
|---|---|---|---|
| **20** | 26.4 | $C_{42}H_{43}NO_9S$ $[M+NH_4]^+$ 755.3 | 755.2 |
| **21** | 31.2 | $C_{58}H_{65}NO_{10}$ $[M+NH_4]^+$ 953.5 | 953.3 |
| **22** | 30.2 | $C_{65}H_{70}N_2O_{13}S$ $[M+NH_4]^+$ 1136.5 | 1136.3 |

**References**


1. Scriabine, I. *Bull. Soc. Chim. Fr.* 1947, 454.
2. Hasegawa, Y.; Shindou, S.; Hattori, T.; Obata, T.; Horiuchi, F.; Hayakawa, H.; Kumazawa, H. Preparation of Pyridylmethylcarbamoylethenylbenzene Derivatives as Immunomodulators and Therapeutic Agents for Nephrotic Syndrome, Circulatory and Respiratory Diseases WO Patent, 2000042013, June 20, 2000.
3. Norris, J. F.; Ware, V. W. *J. Am. Chem. Soc.* **1939**, *61*, 1418.
4. M. Gude, M.; Ryf, J.; White, P. D. *Lett. Pept. Sci.*, **2002**, *9*, 203.

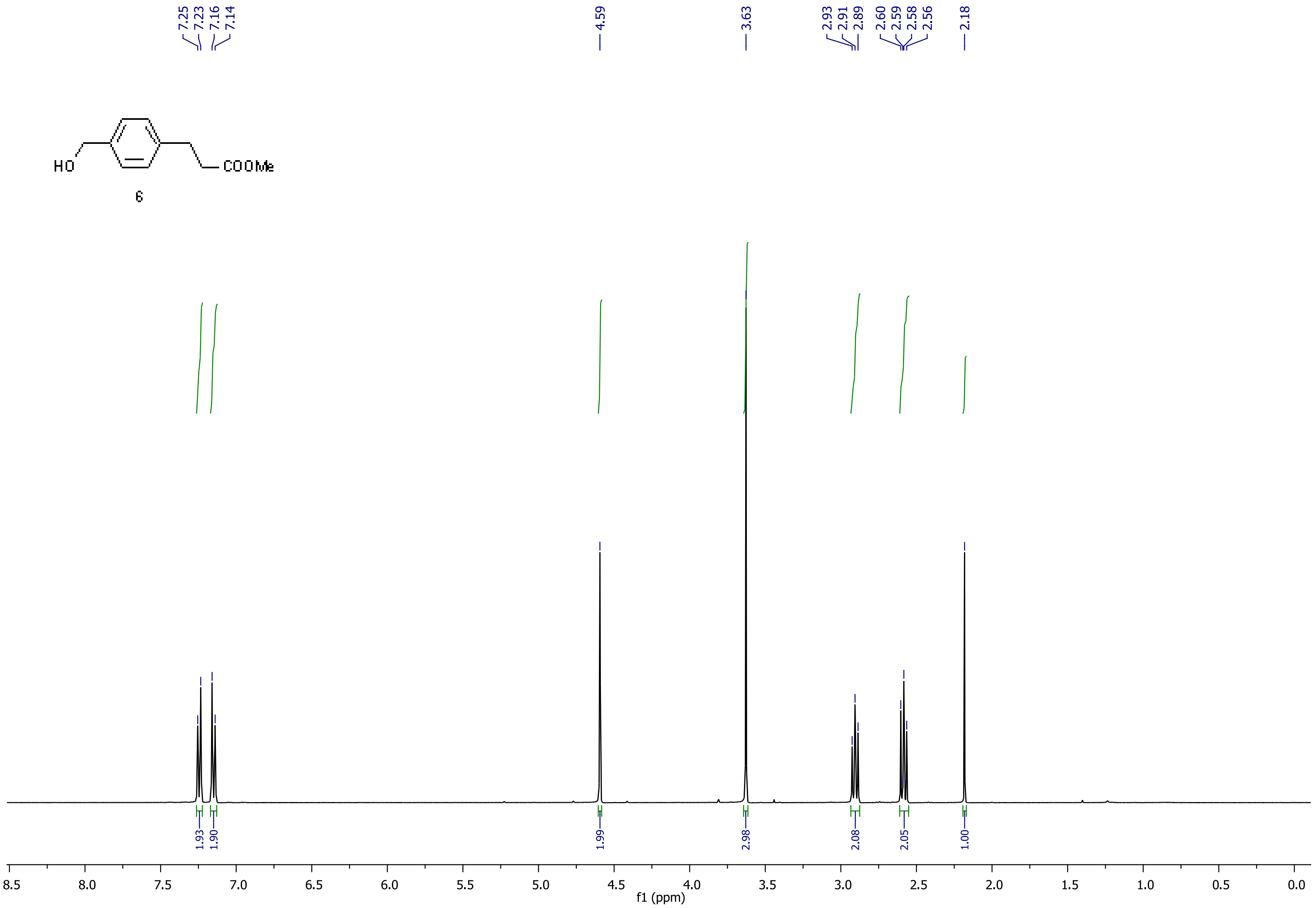
7.25
7.23
7.16
7.14
4.59
3.63
2.93
2.91
2.89
2.60
2.59
2.58
2.56
2.18
HO
COOMe
6
1.93
1.90
1.99
2.98
2.08
2.05
1.00
8.5
8.0
7.5
7.0
6.5
6.0
5.5
5.0
4.5
4.0
3.5
3.0
2.5
2.0
1.5
1.0
0.5
0.0
f1 (ppm)

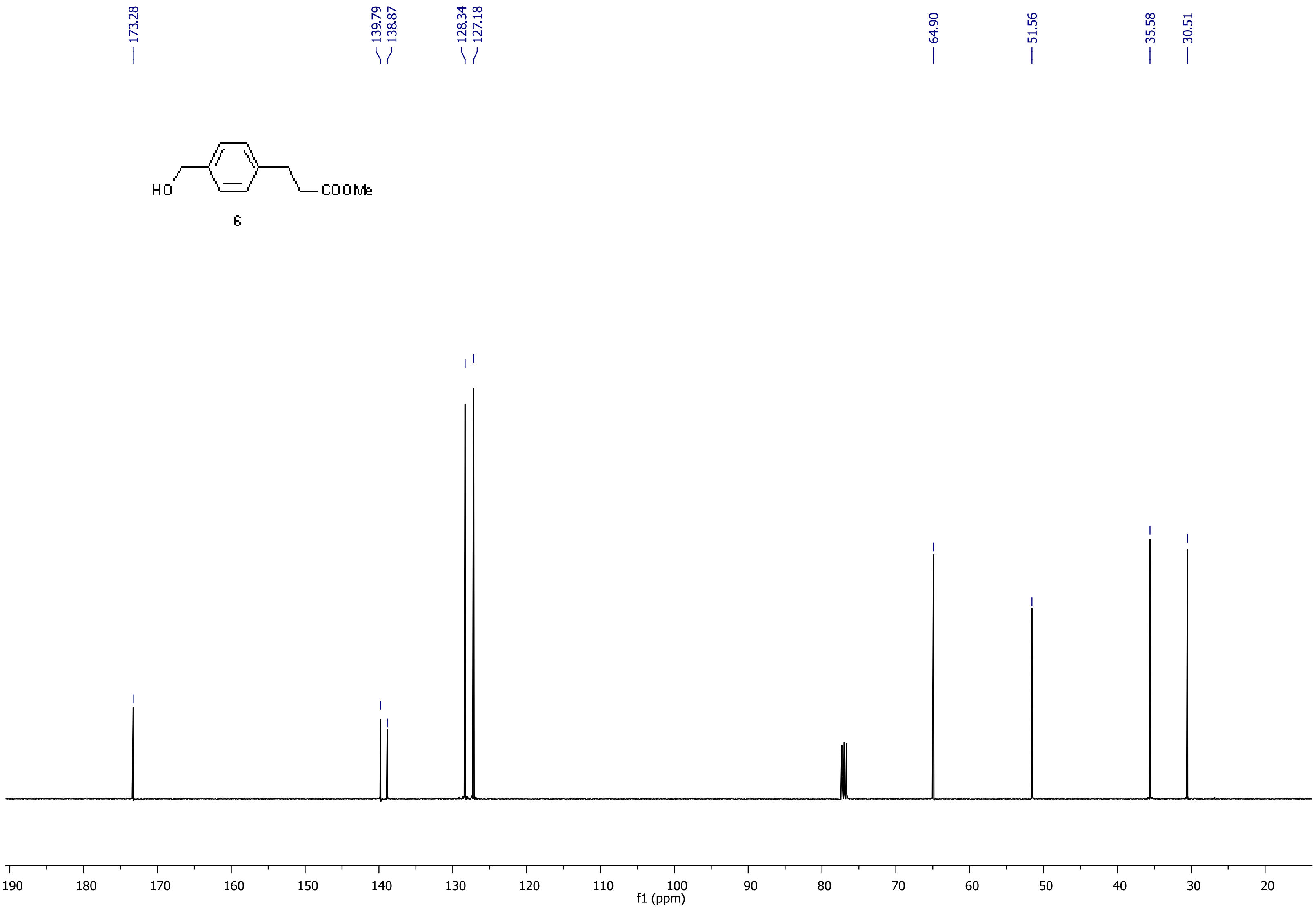
173.28
139.79
138.87
128.34
127.18
64.90
51.56
35.58
30.51
HO
COOMe
6
190
180
170
160
150
140
130
120
110
100
90
80
70
60
50
40
30
20
f1 (ppm)

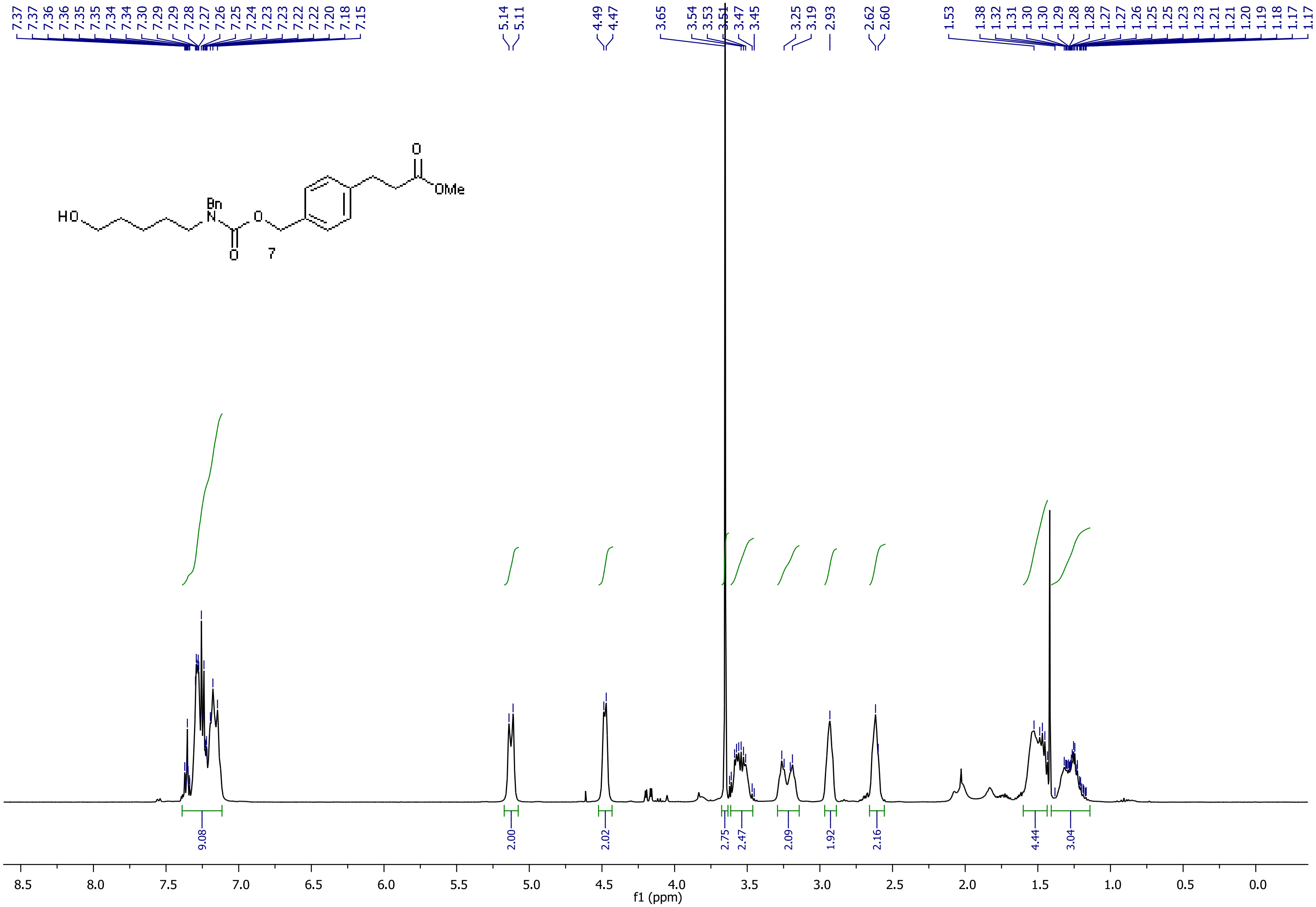

HO
Bn
N
O
O
OMe
7
9.08
2.00
2.02
2.75
2.47
2.09
1.92
2.16
4.44
3.04
f1 (ppm)

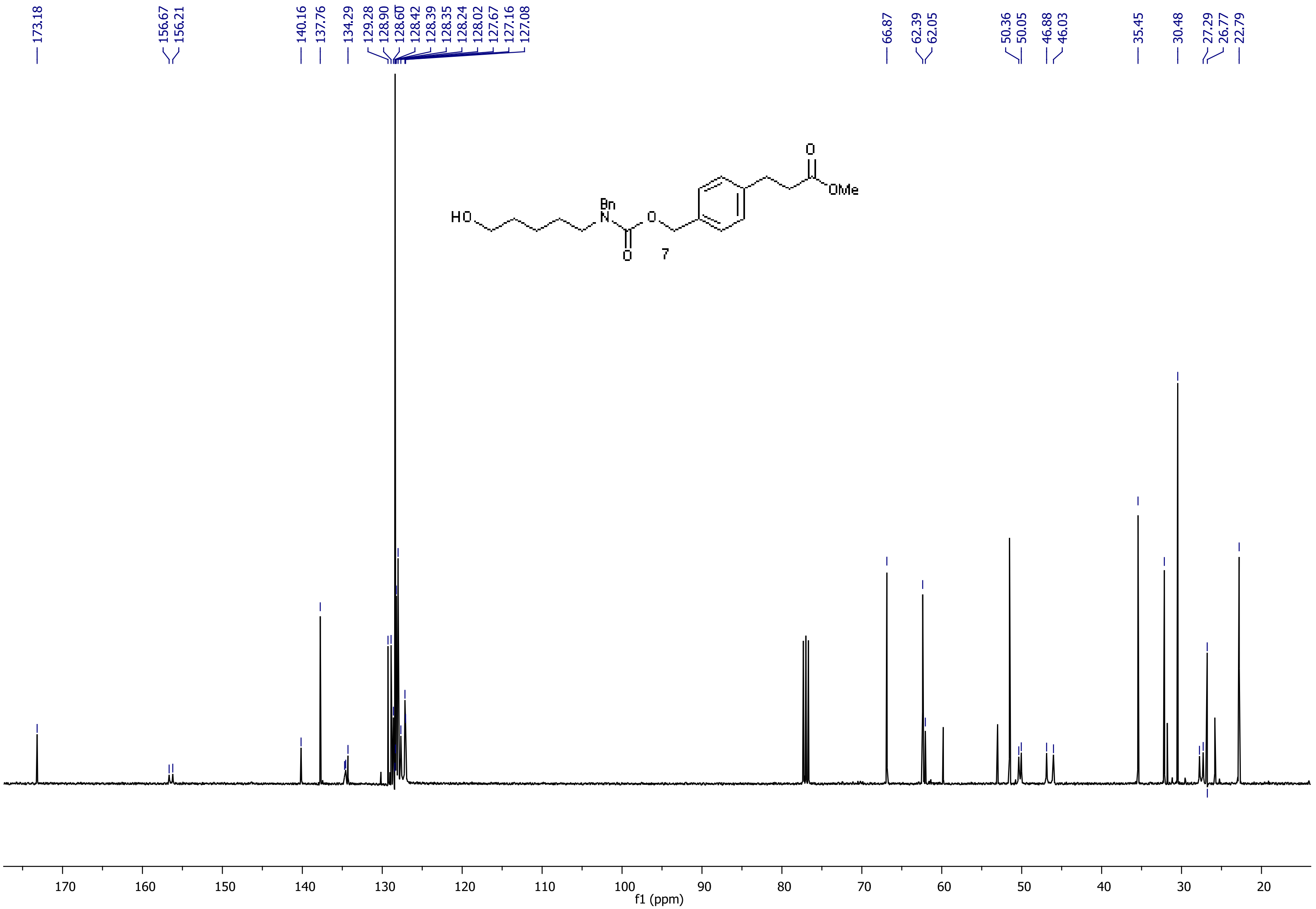

173.18
156.67
156.21
140.16
137.76
134.29
129.28
128.90
128.60
128.42
128.39
128.35
128.24
128.02
127.67
127.16
127.08
66.87
62.39
62.05
50.36
50.05
46.88
46.03
35.45
30.48
27.29
26.77
22.79
HO
Bn
N
O
O
O
OMe
7
f1 (ppm)

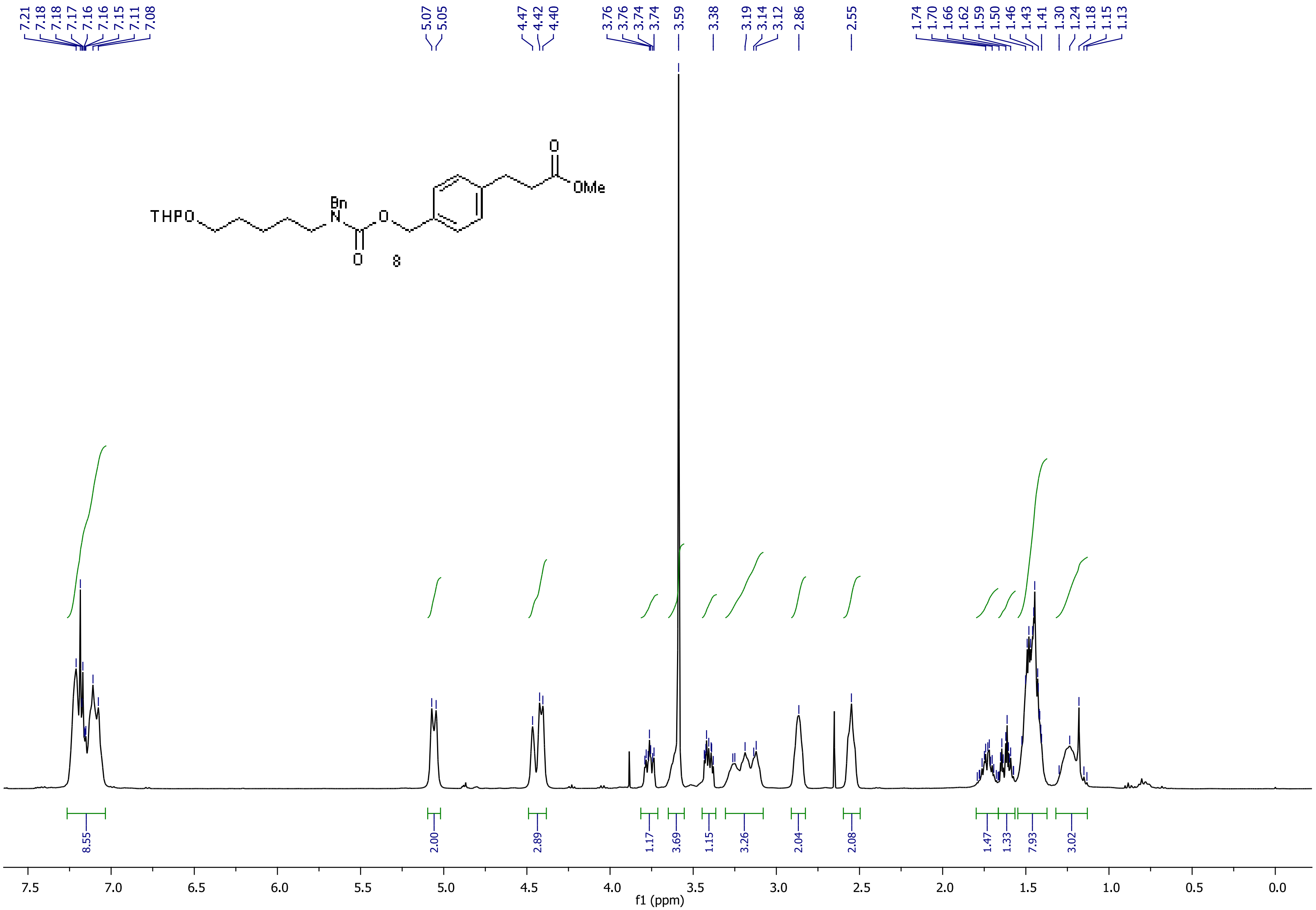
7.21 7.18 7.18 7.17 7.16 7.16 7.15 7.11 7.08
5.07 5.05
4.47 4.42 4.40
3.76 3.76 3.74 3.74
3.59
3.38
3.19 3.14 3.12
2.86
2.55
1.74 1.70 1.66 1.62 1.59 1.50 1.46 1.43 1.41
1.30 1.24 1.18 1.15 1.13
THPO
Bn
N
O
O
O
OMe
8
8.55
2.00
2.89
1.17
3.69
1.15
3.26
2.04
2.08
1.47
1.33
7.93
3.02
7.5 7.0 6.5 6.0 5.5 5.0 4.5 4.0 3.5 3.0 2.5 2.0 1.5 1.0 0.5 0.0
f1 (ppm)

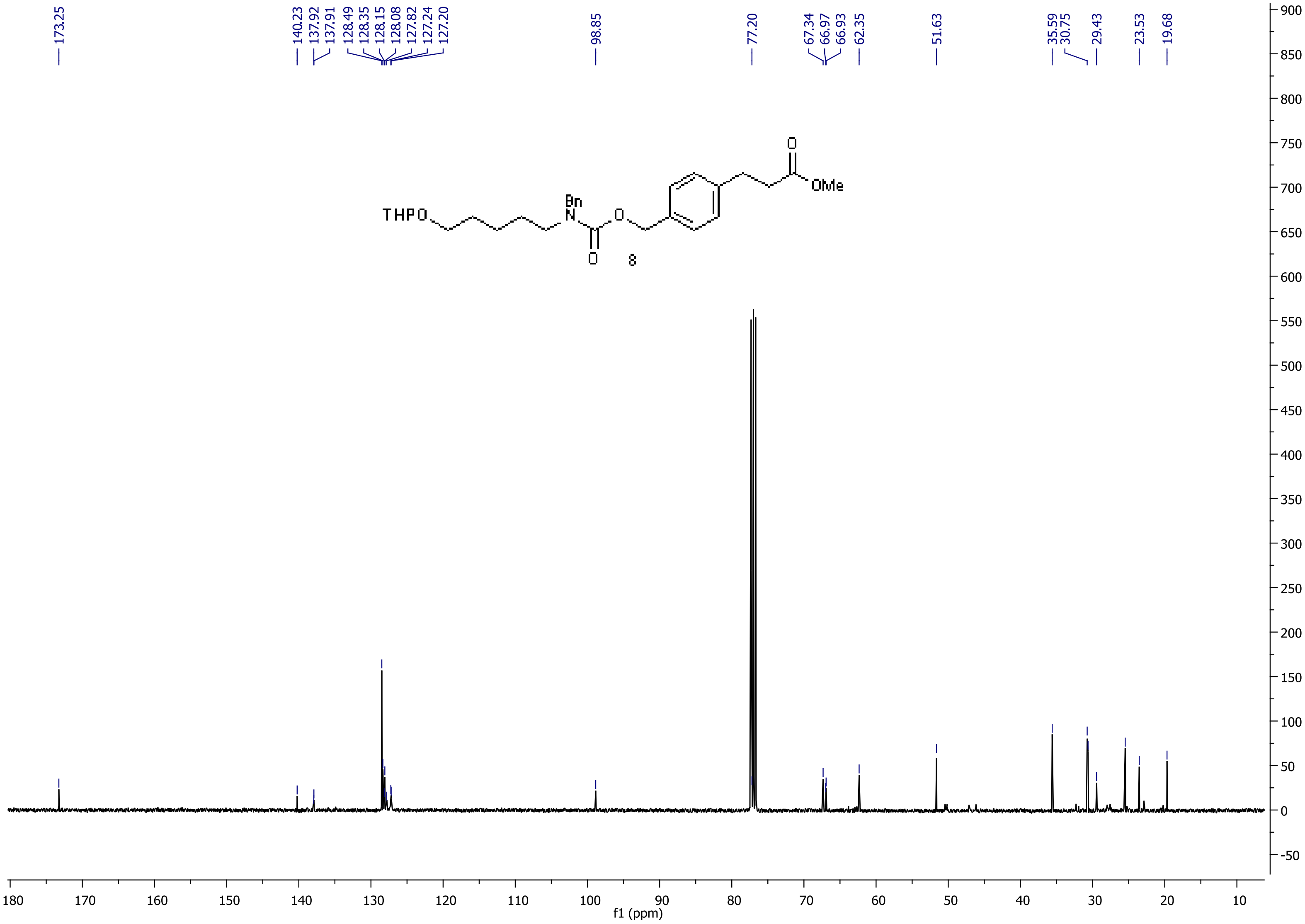

173.25
140.23
137.92
137.91
128.49
128.35
128.15
128.08
127.82
127.24
127.20
98.85
77.20
67.34
66.97
66.93
62.35
51.63
35.59
30.75
29.43
23.53
19.68
THPO
Bn
N
O
OMe
8
f1 (ppm)

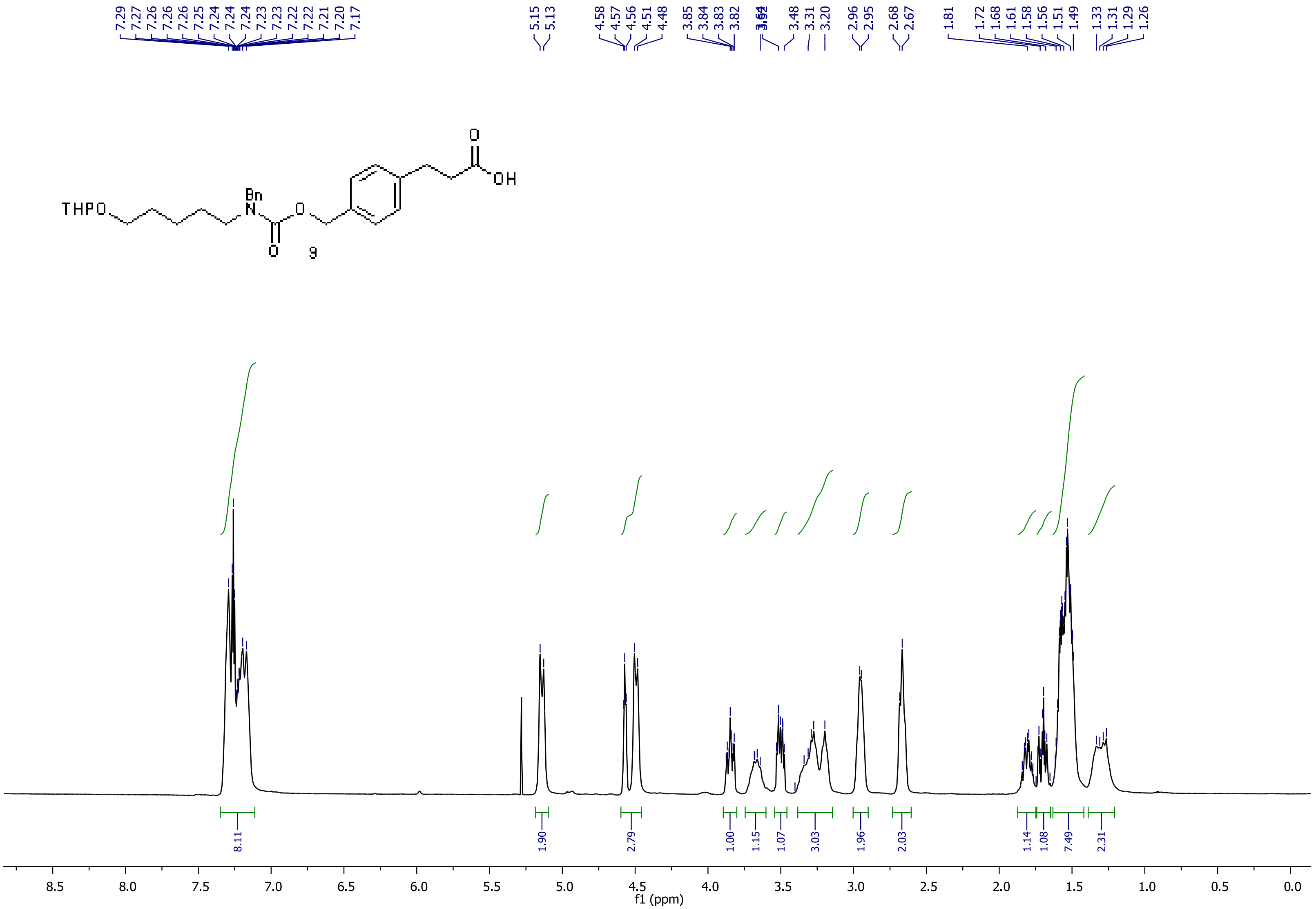

7.29 7.27 7.26 7.26 7.25 7.24 7.24 7.23 7.23 7.22 7.22 7.21 7.20 7.17
5.15 5.13
4.58 4.57 4.56 4.51 4.48
3.85 3.84 3.83 3.82
3.48 3.31 3.20
2.96 2.95 2.68 2.67
1.81 1.72 1.68 1.61 1.58 1.56 1.51 1.49 1.33 1.31 1.29 1.26
THPO
Bn
N
O
OH
9
8.11 1.90 2.79 1.00 1.15 1.07 3.03 1.96 2.03 1.14 1.08 7.49 2.31
8.5 8.0 7.5 7.0 6.5 6.0 5.5 5.0 4.5 4.0 3.5 3.0 2.5 2.0 1.5 1.0 0.5 0.0
f1 (ppm)

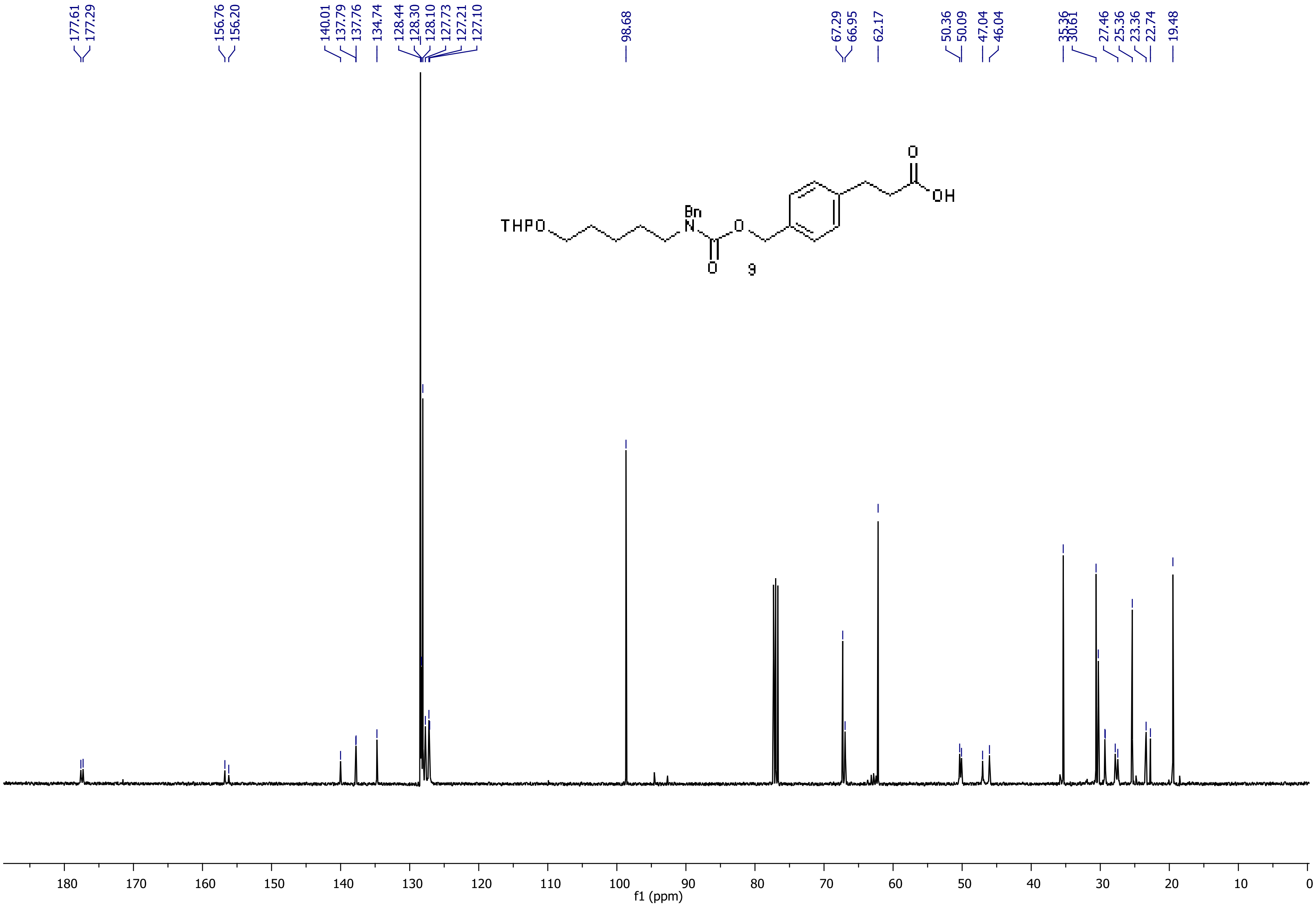

177.61
177.29
156.76
156.20
140.01
137.79
137.76
134.74
128.44
128.30
128.10
127.73
127.21
127.10
98.68
67.29
66.95
62.17
50.36
50.09
47.04
46.04
35.36
30.61
27.46
25.36
23.36
22.74
19.48
THPO
Bn
N
O
O
OH
O
9
180 170 160 150 140 130 120 110 100 90 80 70 60 50 40 30 20 10 0
f1 (ppm)

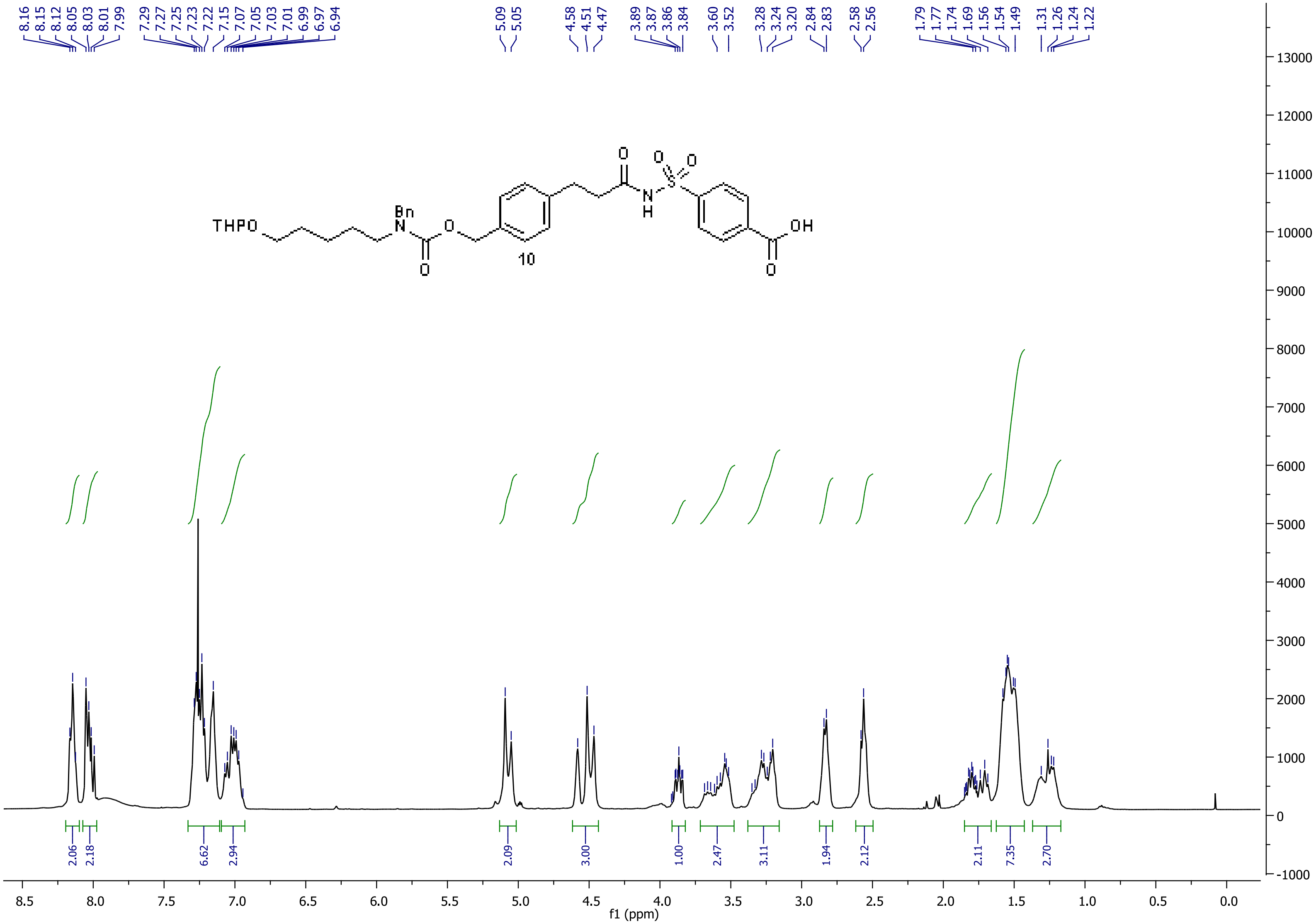

THPO
Bn
N
O
10
OH
f1 (ppm)

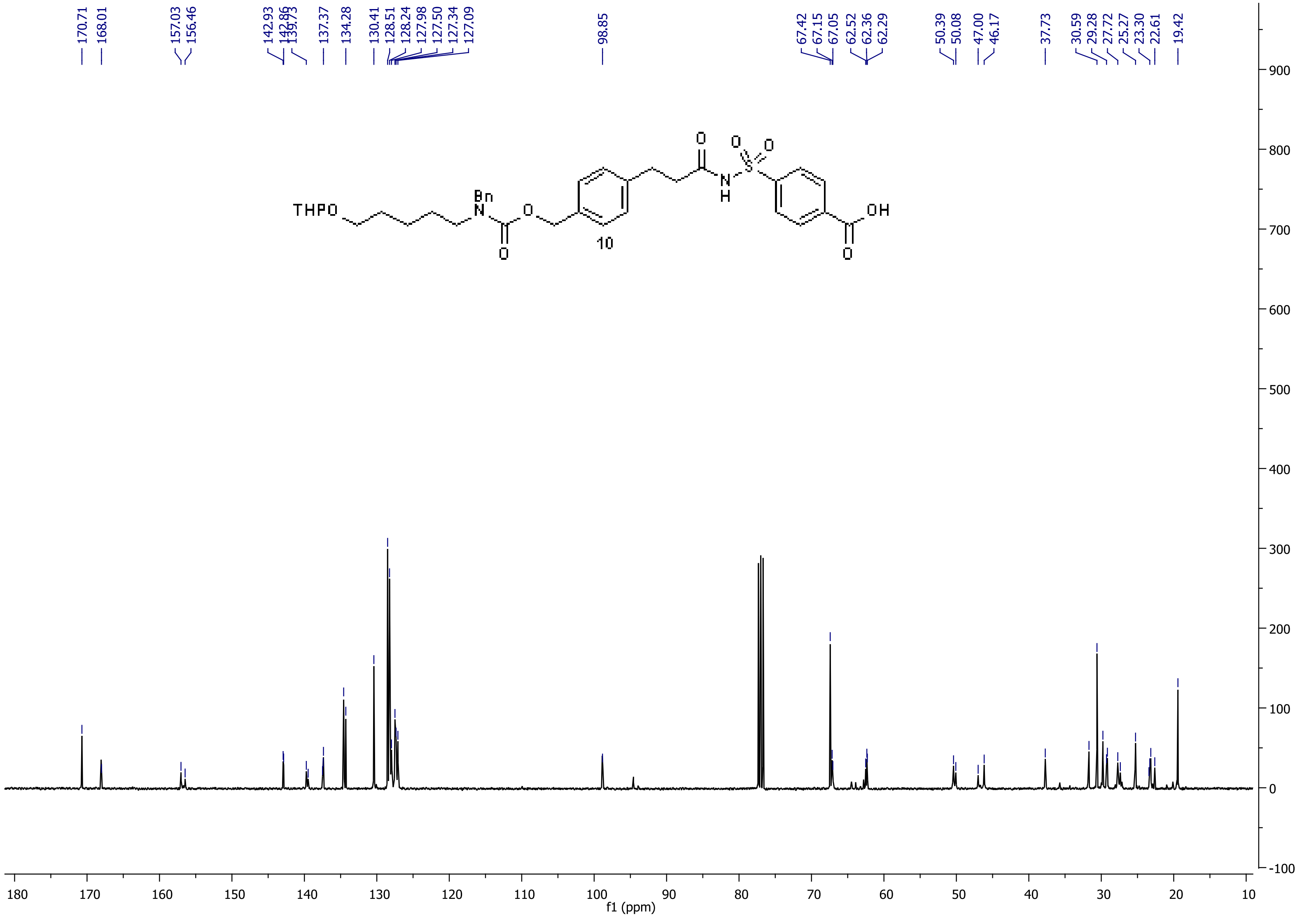
170.71
168.01
157.03
156.46
142.93
142.86
139.73
137.37
134.28
130.41
128.51
128.24
127.98
127.50
127.34
127.09
98.85
67.42
67.15
67.05
62.52
62.36
62.29
50.39
50.08
47.00
46.17
37.73
30.59
29.28
27.72
25.27
23.30
22.61
19.42
THPO
Bn
N
O
O
O
N
H
O
S
O
OH
O
10
f1 (ppm)

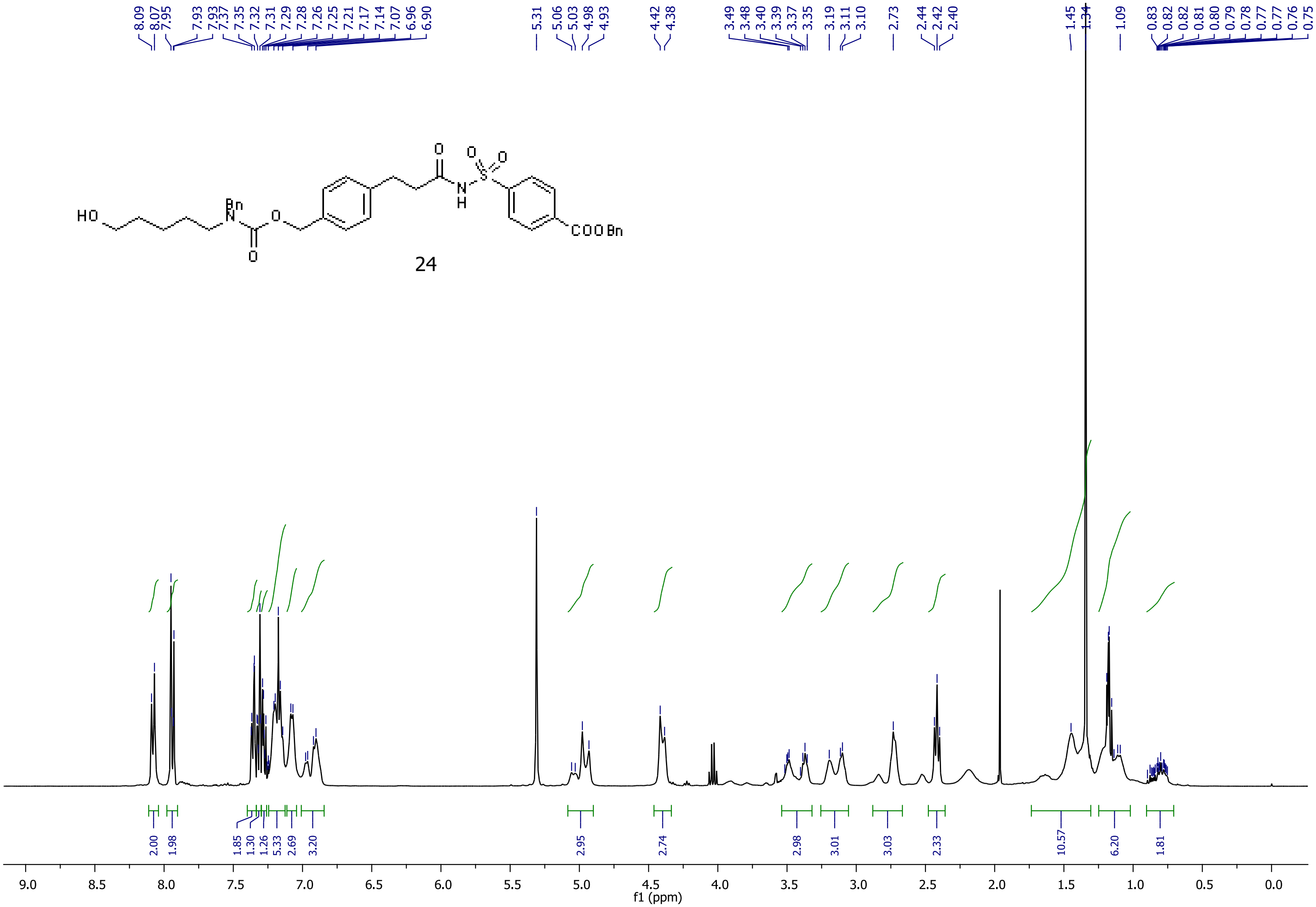
24
f1 (ppm)

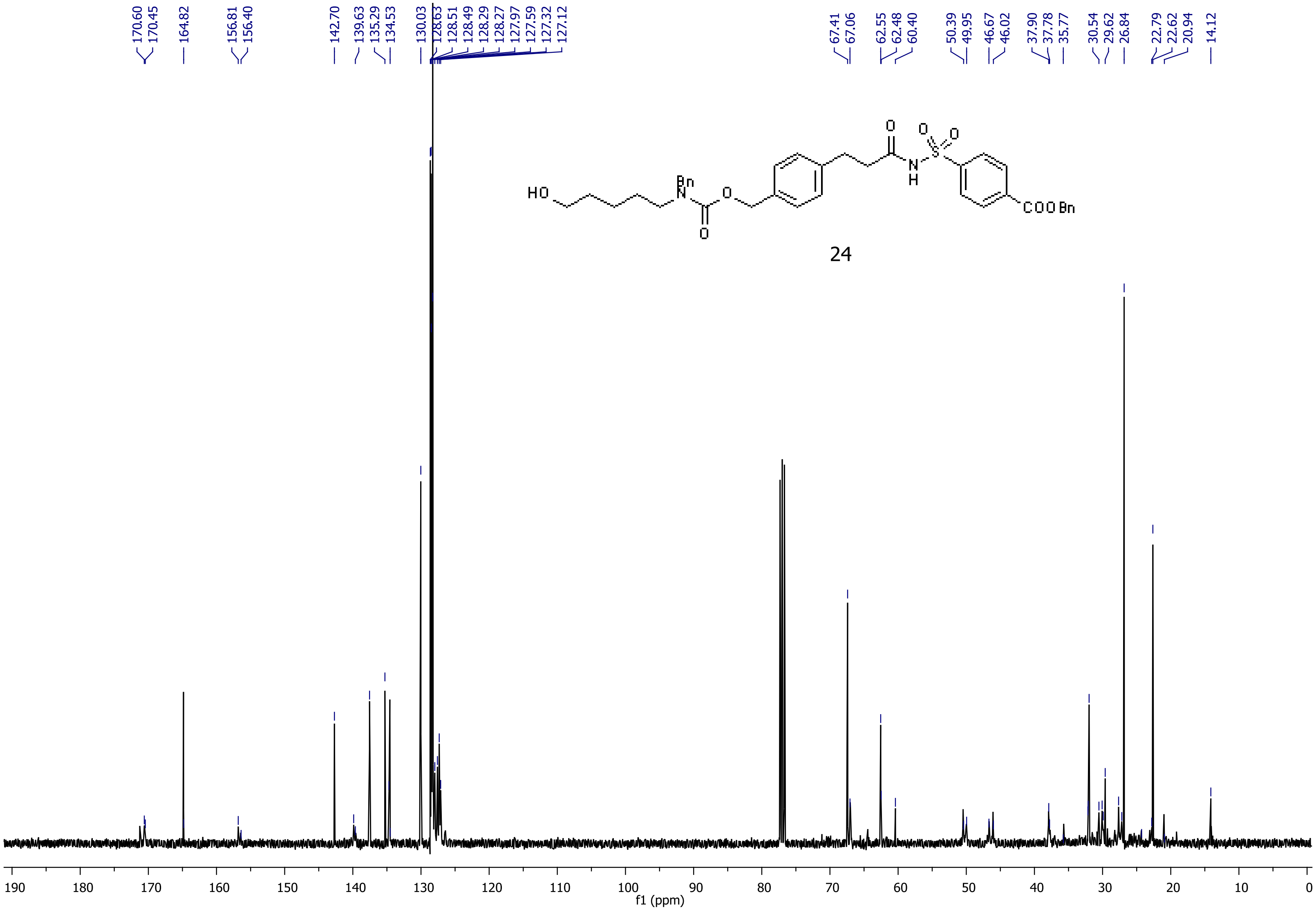
170.60
170.45
164.82
156.81
156.40
142.70
139.63
135.29
134.53
130.03
128.65
128.51
128.49
128.29
128.27
127.97
127.59
127.32
127.12
67.41
67.06
62.55
62.48
60.40
50.39
49.95
46.67
46.02
37.90
37.78
35.77
30.54
29.62
26.84
22.79
22.62
20.94
14.12
HO
Bn
N
O
O
O
N
H
S
O
O
COOBn
f1 (ppm)

24

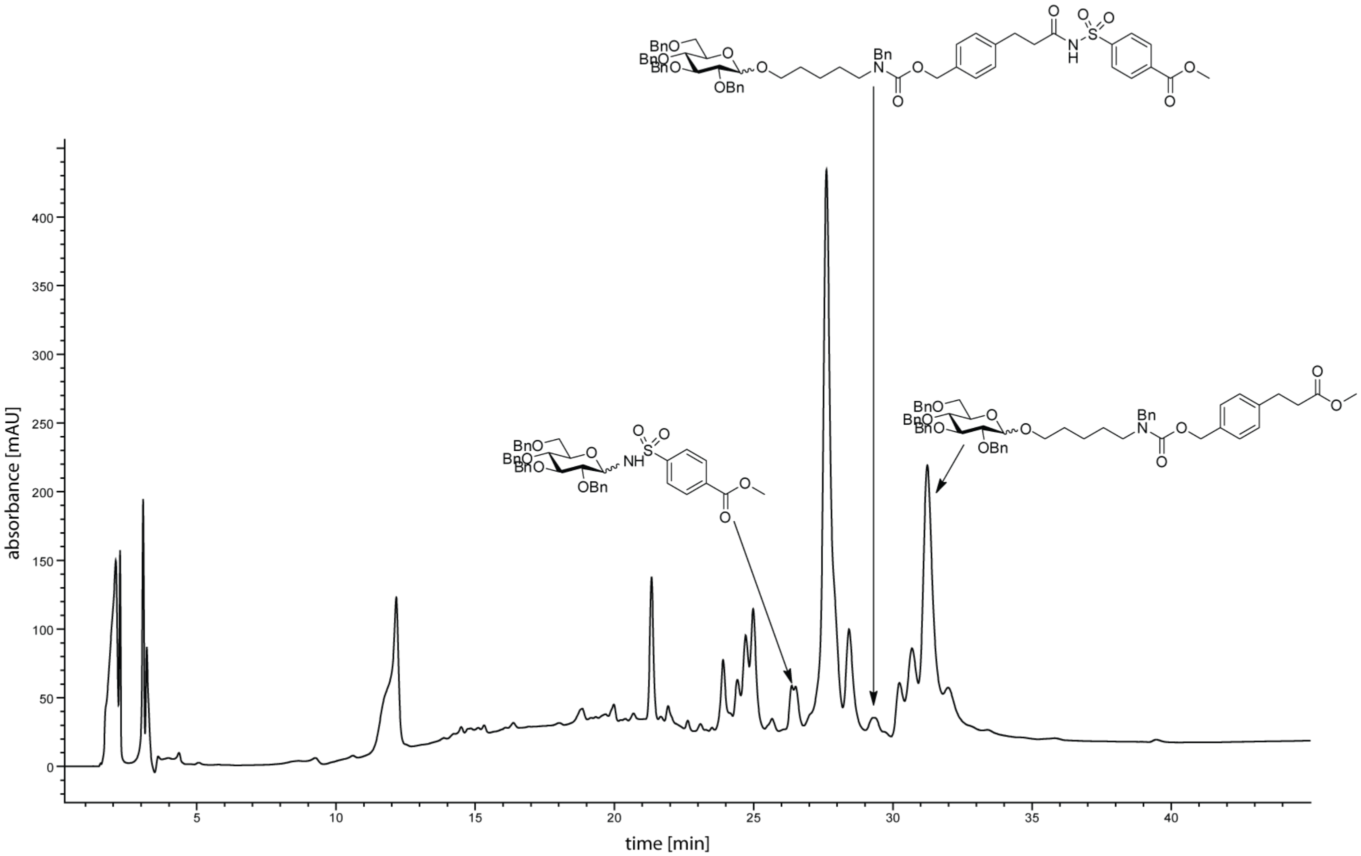


**LC–MS analysis:** Glycosylation was performed with **19** (3 × 3 equiv) and **11** under NIS/TfOH activation and previous washing step with activator solution followed by Zemplén-cleavage reaction; conditions for RP chromatography: column: Nucleosil C4 (120 mm × 5 mm); detection: UV detection at 230 nm; eluents: water (+ 0.1% formic acid) and acetonitrile (+ 0.1% formic acid); gradient: isocratic at 10% for 5 min → 10% → 80% acetonitrile in 30 min → 95% acetonitrile in 10 min.

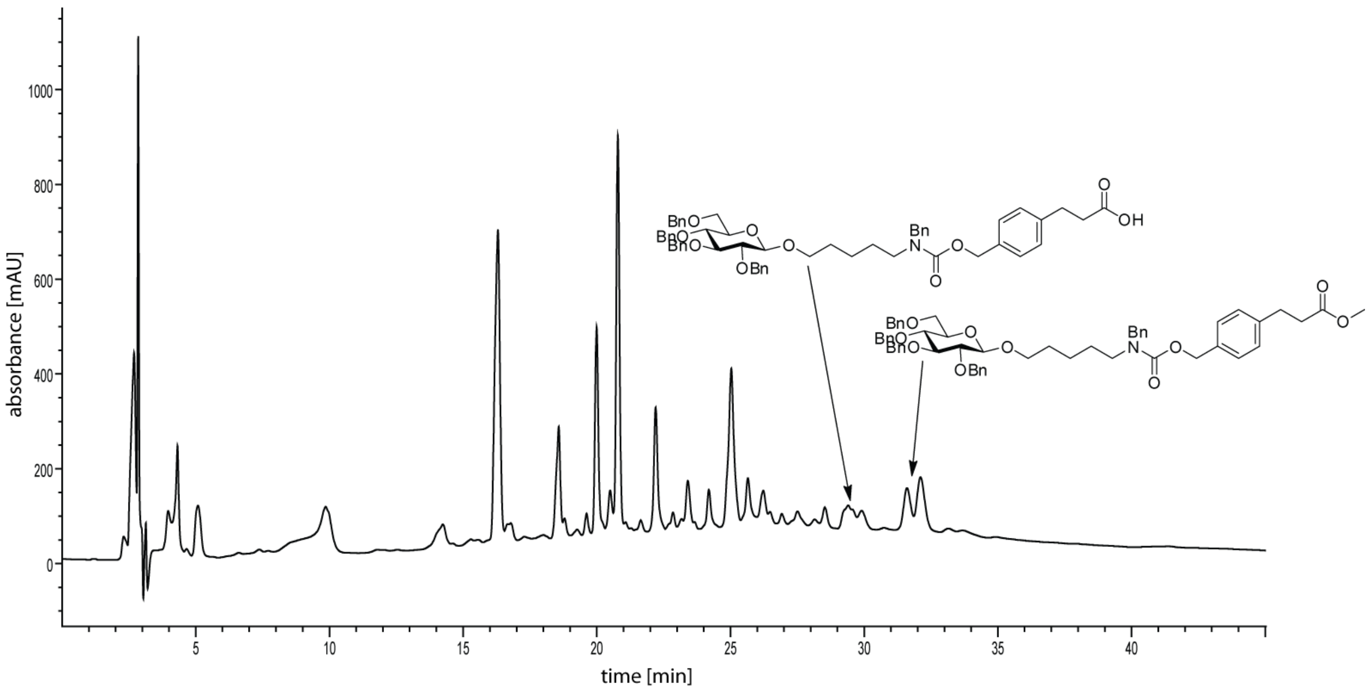


**LC–MS analysis:** Glycosylation was performed with **19** (3 × 3 equiv) and **12** under NIS/TfOH activation and previous washing step with activator solution followed by Zemplén-cleavage reaction; conditions for RP chromatography: column: Nucleosil C4 (120 mm × 5 mm); detection: UV detection at 210 nm; eluents: water (+ 0.1% formic acid) and acetonitrile (+ 0.1% formic acid); gradient: isocratic at 10% for 5 min → 10% → 80% acetonitrile in 30 min → 95% acetonitrile in 10 min.

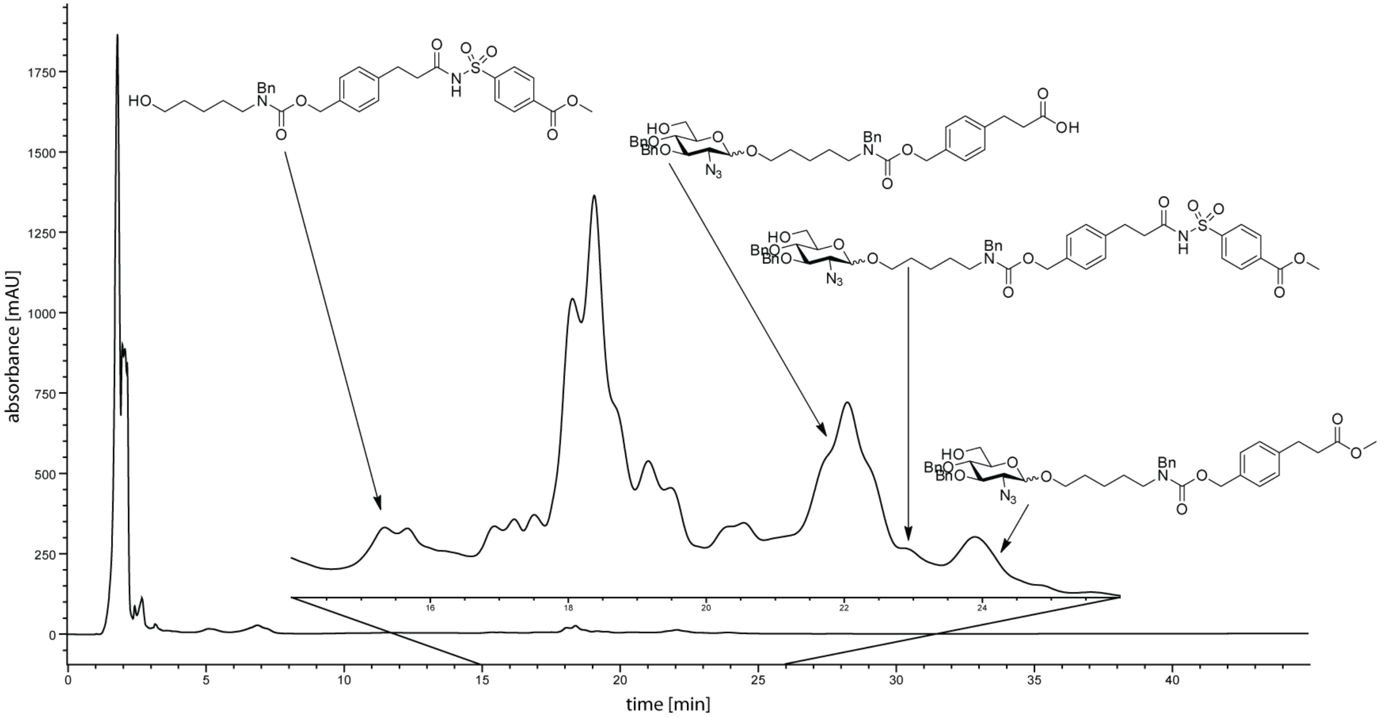


**LC–MS analysis:** Glycosylation was performed with **17** (3 × 1.1 equiv) and **11** under NIS/TfOH activation without previous washing step with activator solution followed by Zemplén-cleavage reaction; conditions for RP chromatography: column: Nucleosil C4 (120 mm × 5 mm); detection: UV detection at 230 nm; eluents: water (+ 0.1% formic acid) and acetonitrile (+ 0.1% formic acid); gradient: isocratic at 10% for 5 min → 10% → 80% acetonitrile in 30 min → 95% acetonitrile in 10 min.

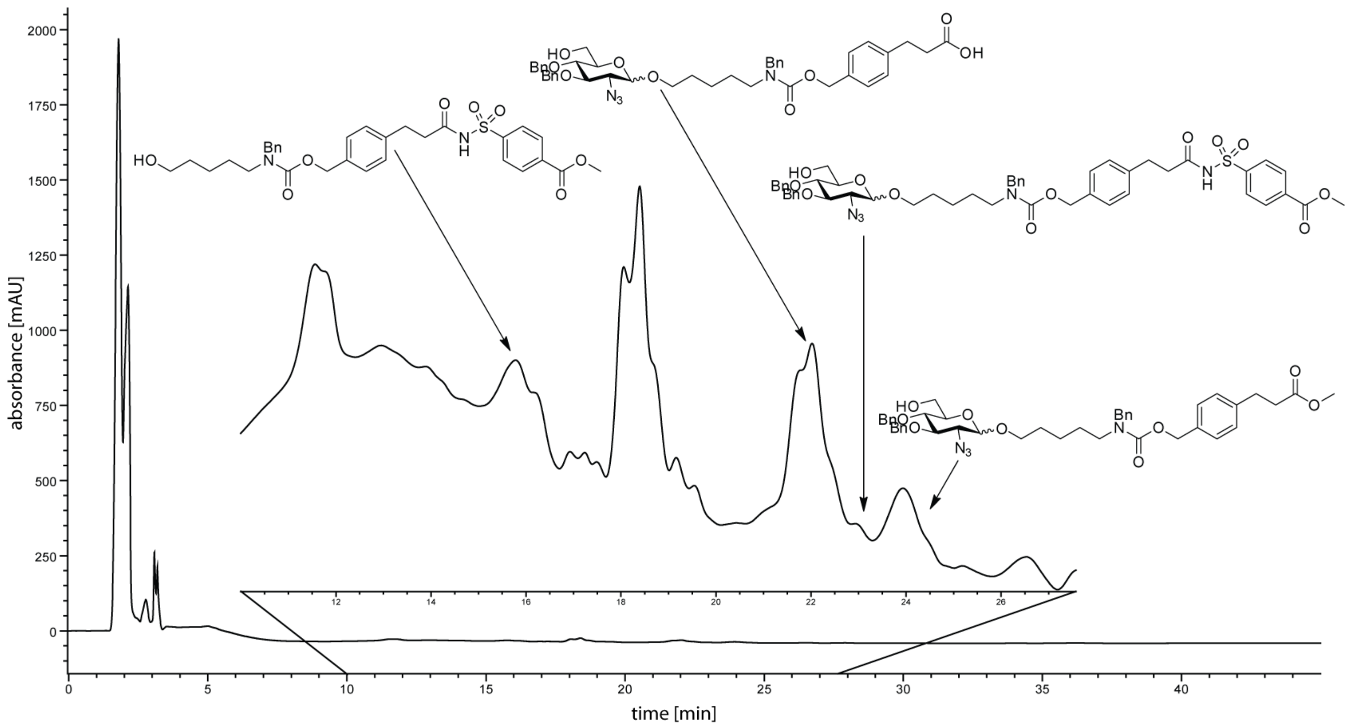


**LC–MS analysis:** Glycosylation was performed with **17** (3 × 1.1 equiv) and **11** under NIS/TfOH activation and previous washing step with activator solution followed by Zemplén-cleavage reaction; conditions for RP chromatography: column: Nucleosil C4 (120 mm × 5 mm); detection: UV detection at 230 nm; eluents: water (+ 0.1% formic acid) and acetonitrile (+ 0.1% formic acid); gradient: isocratic at 10% for 5 min → 10% → 80% acetonitrile in 30 min → 95% acetonitrile in 10 min.

The peaks in the chromatograms are polystyrene (Merrifield resin)-derived compounds with strong UV absorbance. See also: K. Tanaka, Y. Fujii, H. Tokimoto, Y. Mori, S.-i. Tanaka, G.-m. Bao, E. R. O. Siwu, A. Nakayabu and K. Fukase, *Chem. Asian J.*, 2009, **4**, 574. and the related supporting information.

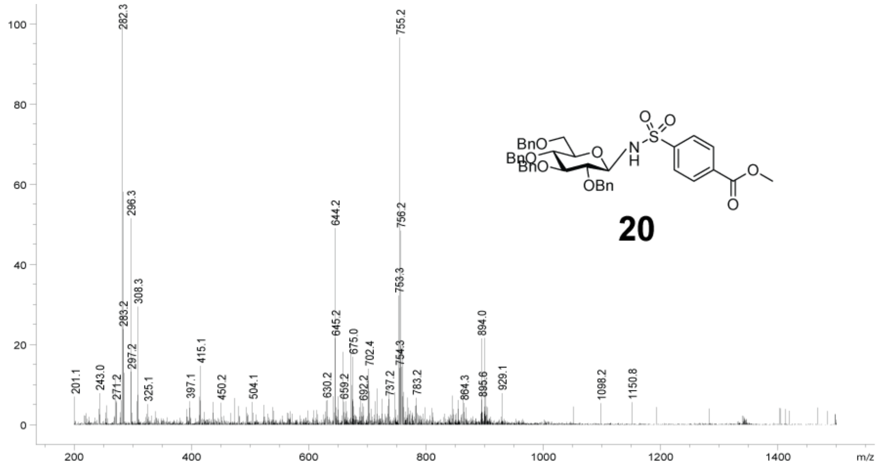


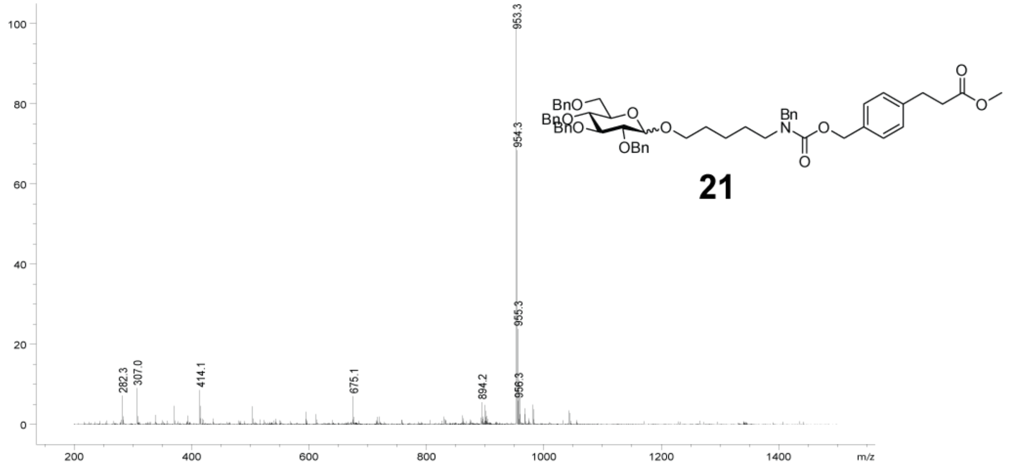


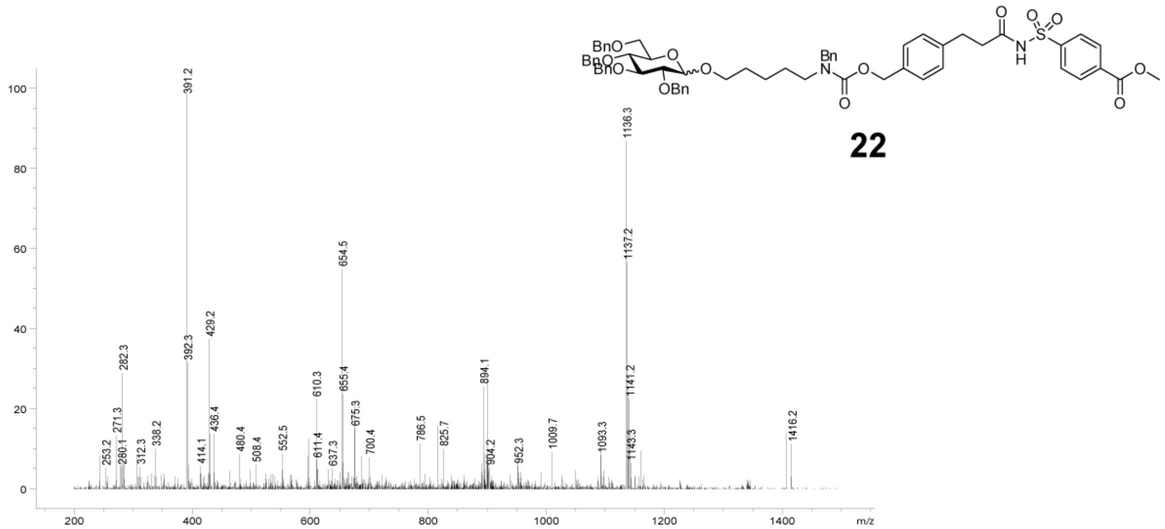


**MS spectra**: compounds **20**–**22** (taken from RPHPLC–MS analysis).
